# A new strength assessment to evaluate the association between muscle weakness and gait pathology in children with cerebral palsy

**DOI:** 10.1371/journal.pone.0191097

**Published:** 2018-01-11

**Authors:** Marije Goudriaan, Angela Nieuwenhuys, Simon-Henri Schless, Nathalie Goemans, Guy Molenaers, Kaat Desloovere

**Affiliations:** 1 KU Leuven–University of Leuven, Department of Rehabilitation Sciences, Neuromotor Rehabilitation Research Group, Leuven, Belgium; 2 KU Leuven–University of Leuven, Department of Development and Regeneration, Organ Systems, Leuven, Belgium; 3 University Hospitals Leuven, Department of Child Neurology, Leuven, Belgium; 4 University Hospitals Leuven, Department of Orthopedics, Clinical Motion Analysis Laboratory (CERM), Pellenberg, Belgium; Boston Children's Hospital / Harvard Medical School, UNITED STATES

## Abstract

**Aim:**

The main goal of this validation study was to evaluate whether lower limb muscle weakness and plantar flexor rate of force development (RFD) related to altered gait parameters in children with cerebral palsy (CP), when weakness was assessed with maximal voluntary isometric contractions (MVICs) in a gait related test position. As a subgoal, we analyzed intra- and intertester reliability of this new strength measurement method.

**Methods:**

Part 1 –Intra- and intertester reliability were determined with the intra-class correlation coefficient (ICC_2,1_) in 10 typical developing (TD) children (age: 5–15). We collected MVICs in four lower limb muscle groups to define maximum joint torques, as well as plantar flexor RFD.

Part 2 –Validity of the strength assessment was explored by analyzing the relations of lower limb joint torques and RFD to a series of kinematic- and kinetic gait features, the GDI (gait deviation index), and the GDI-kinetic in 23 children with CP (GMFCS I-II; age: 5–15) and 23 TD children (age: 5–15) with Spearman’s rank correlation coefficients.

**Results:**

Part 1 –The best reliability was found for the torque data (Nm), with the highest ICC_2,1_ (0.951) for knee extension strength (inter) and the lowest (0.693) for dorsiflexion strength (intra). For plantar flexor RFD, the most reliable window size was 300 milliseconds (ICC_2,1_: 0.828 (inter) and 0.692 (intra)).

Part 2 –The children with CP were significantly weaker than the TD children (p <0.001). Weakness of the dorsiflexors and plantar flexors associated with delayed and decreased knee flexion angle during swing, respectively. No other significant correlations were found.

**Conclusion:**

While our new strength assessment was reliable, intra-joint correlations between weakness, RFD, and gait deviations were low. However, we found inter-joint associations, reflected by a strong association between plantar- and dorsiflexor weakness, and decreased and delayed knee flexion angle during swing.

## Introduction

Cerebral palsy (CP) is the most common physical disability in childhood with a prevalence of 2–3 in 1000 live born infants [1,2]. Children with CP have varying motor deficits, including neuromuscular symptoms, such as a shortened muscle-tendon unit, spasticity, lack of selective muscle control, and muscle weakness [2–4]. These symptoms adversely affect normal development of functional activities such as walking [3]. Many treatment modalities are focusing on these neuromuscular symptoms, aiming to improve gait in children with CP [3]. Therefore, a good insight into the interaction between these neuromuscular symptoms and gait may have a significant impact on the clinical decision-making process. Since muscle weakness, as one of these symptoms, is considered to be a major interfering factor on gait [5], there has been a clinical interest in the association between muscle weakness and pathological gait features.

The relationship between muscle weakness and gait deviations in children with CP has been analyzed by several researchers, but their results are difficult to compare and sometimes contradictory [6–15]. The main problems are the methodological differences, due to the variety of applied strength measurement devices, test positions during the weakness assessments, and selected parameters extracted from the weakness assessment and from 3D gait analysis (Supplementary materials: S1 Table).

A maximal voluntary isometric contraction (MVIC) measured with a hand-held dynamometer is a rather simple, relatively cheap, and easy accessible way to assess muscle weakness in children with CP and the overall reliability is considered to be good [16–21] (Supplementary materials: S2 Table). However, in all these reliability studies, the strength of the assessor had an influence on the measurement outcomes, plus compensatory movements of the participants during the measurements could not be excluded [16–21]. Further, in previous studies analyzing the effect of muscle weakness on gait, the test positions of the weakness assessment appear to be selected independently from the joint angles (and thus muscle lengths) observed during gait [6–15]. By selecting a test position that mimics the averaged joint angles of gait, the relationship between MVIC outcomes and gait parameters may be improved. Also, due to the changes in motor control and muscle morphology [4,22], not only the maximal net joint torques [6–8,11–13,15], but rate of force development (RFD) could be a relevant parameter that influences functional performance as well [23]. This is especially the case for specific gait phases that are characterized by high angular velocities, such as the push-off around the ankle joint.

Most of the extracted kinematic- and kinetic gait parameters in previous literature were linked to specific gait cycle phases during which the evaluated muscles were assumed to be active [6,9–15]. The rationale behind these study designs was that weakness of a certain muscle group would be related to an altered joint angle or net joint torque delivered by that muscle group. However, a reduced ankle torque while walking at self-selected speed is frequently achieved by reducing the external lever arm, i.e. keeping the ground reaction force closely aligned to the joint center the muscle is acting on. These compensations for weakness at specific joints also cause kinematic- and kinetic changes in other joints. Therefore, a gait deviation index describing the entire kinematic- and kinetic gait pattern might be another interesting parameter to explore, when analyzing the relationship between pathological gait and muscle weakness [24–26].

To summarize, hand-held dynamometry seems appropriate to quantify weakness of lower limb muscle groups, but compensation mechanisms and influence of assessor strength were not taken into consideration in previous studies. Further, the test positions used during the weakness assessments were not related to gait. Finally, the selected parameters from both weakness assessments and gait appear to be incomplete.

Therefore, the main goal of this study was to determine the validity of the new strength assessment by assessing the relationship between muscle weakness and the altered gait parameters in children with CP, when the above-mentioned limitations were minimized. As a subgoal, reliability of our new strength measurement was also analyzed. Muscle weakness was assessed when the participants and the dynamometer were fixed in a custom-made chair. The test position was based on the average joint angles of gait. The outcomes of the weakness assessment were compared with a series of kinematic and kinetic gait features, as well as gait deviation indices, and walking speed. A detailed overview of these study goals and our hypotheses can be found in Table 1.

**Table 1:** Study design.

**Study design**			
**Study goal.** To determine the validity of the new strength assessment by assessing the relationship between muscle weakness and the altered gait parameters in children with CP. The children were positioned in a more gait related test position, while limiting the influence of assessor strength, and compensation mechanisms. Additionally, we determined the reliability of our new weakness assessment. First, intra- and intertester reliability were determined (part 1). Secondly, the validity of new strength measurement was explored, by analyzing the association between the weakness outcomes and several gait parameters (part 2).			
**Part 1 –Reliability of strength assessment**		**Part 2 –Validity of the strength assessment**	
Hypotheses regarding the reliability of the new strength assessment: 1. By fixing the children and the dynamometer assessor strength and compensation mechanism will be decreased, resulting similar reliability outcomes for intra- and intertester measurements. 2. Averaging the MVIC outcomes and RFD values will have better reliability results compared to the absolute maximum values.		Hypotheses regarding the validity of the strength assessment: 3. Children with CP have lower maximal net joint torques and plantar flexor RFD during the MVICs. 4. High correlations are found between MVIC outcomes and the gait parameters when MVICs are measured with the new strength measurement method. 5. Highest correlations are found between plantar flexion RFD and power generation at the ankle, and between MVIC outcomes, and the GDI and GDI-kinetic.	
**Subjects**			
10 TD children, aged between 5–15 years		23 children with CP, aged between 5–15 years 23 TD, aged between 5–15 years	
**Data collection**			
**Intra-tester measurements** N:5 (left + right)	**Intertester measurements** N:5 (left + right)	**MVIC measurements** N: 23 children with CP 23 TD children	**3D gait analyses** N: 23 children with CP 23 TD children
MVICs of knee extensors, knee flexors, dorsiflexors, and plantar flexors		MVICs of knee extensors, knee flexors, dorsiflexors, and plantar flexors	Walking at two walking speeds
**Data analysis**			
**Intra- and intertester data.** Absolute maximal- and averaged values over three trials.		**MVICs.** Maximal normalized net joint torque (Nm/kg) for all four muscle groups RFD of the plantar flexors in Newtons per second (N/s) **Gait (both walking speeds).** Non-dimensional walking speed. Seven kinematic and five kinetic features. GDI and GDI-kinetic	
**Statistical analysis**			
**Hypotheses 1–2.** ICC_2,1_, CI, SEM, MDD and F-value of Anova for intra- and intertester measurements.		**Hypotheses 3.** Mann-Whitney U test to determine differences between children with CP and TD children. **Hypothesis 4–5.** Spearman’s rank correlation between MVICs and plantar flexor RFD, and the gait parameters, GDI and GDI-kinetic.	

Abbreviations in alphabetic order: = CI confidence interval; CP = cerebral palsy; GDI = gait deviation index; ICC = intra-class correlation coefficient; MDD = minimal detectable difference; Nm/kg = Newton meters per kilogram bodyweight; N/s = newtons per second; RFD = rate of force development; SEM = standard error of measurement; TD = typical developing

## Materials and methods

### Part 1: Reliability of the strength assessment

#### Subjects

We conducted a power analysis to determine the sample size. Based on the classification of Fleiss [27], minimal ICC-value (ρ_0_) was set at 0.50 (fair to good) and maximal ICC-value (ρ_1_) at 0.90 (excellent). With an α err prob = 0.05 and power (1-β err prob) = 0.80, this resulted in a minimal sample size of nine participants per reliability assessment (intra and inter)[28].

We recruited 10 TD children, between five and 15 years old without any neurological or neuromuscular problems (Table 2). This study was approved by our local ethics committee (Commissie Medische Ethiek KU Leuven; S56041) and written informed consent was obtained from next of kin, caretakers, or guardians on behalf of the children in accordance with the Declaration of Helsinki. Children aged 12 years or older, signed the informed consent forms themselves as well.

**Table 2:** Subject characteristics of the TD children participating in part 1 of this study.

TD	Gender	Age *years*	Weight *kilogram*	Height *meters*	Assessor *measurement 1*	Assessor *measurement 2*
**Intra-tester**						
TD1	Girl	8.70	27.3	1.31	PTs1	PTs1
TD2	Boy	8.32	25.6	1.32	PTs1	PTs1
TD3	Boy	11.33	35.7	1.49	PTs1	PTs1
TD4	Boy	11.74	36.2	1.48	PTs1	PTs1
TD5	Girl	8.29	29.6	1.29	PTs1	PTs1
**Inter-tester**						
TD6	Girl	11.29	33.1	1.44	PT	PTs2
TD7	Girl	8.56	28.7	1.36	PT	PTs2
TD8	Girl	8.56	26.5	1.33	PT	PTs2
TD9	Boy	9.36	36.3	1.46	PT	PTs2
TD10	Boy	14.37	62.8	1.71	PT	PTs2

Abbreviations in alphabetic order: PT = physical therapist; PTs: physical therapy student; TD = typical developing.

#### Data collection

MVICs were collected bilaterally of knee extensors, knee flexors, dorsiflexors, and plantar flexors in a custom-designed chair, holding the hip, knee and ankle in a position (hip and knee in 30^0^ flexion, ankle in neutral) resembling the average joint angles observed during gait (S1–S3 Figs). These average joint angles were derived from the retrospective baseline 3D gait analysis data from 53 children with CP (average age ± standard deviation: 6.1 ± 2.3) collected at CMAL-Pellenberg and published online [29]. The averaged sagittal joint angles from this retrospective dataset were (average joint angles ± standard deviation): 21.4^0^ ± 7.2^0^ (hip), 26.9 ± 9.9^0^ (knee), and 3.1^0^ ± 9.2^0^ (ankle).

To limit the influence of assessor strength and to decrease compensation mechanisms, the dynamometer and the children were fixed in the chair. The children were secured with a strap over the pelvis and upper legs, and during all measurements the arms were crossed in front of their chest. For the dorsiflexion MVIC, the foot was placed in a heel cuff. A total of three testers, a physical therapist (PT) and two physical therapy master students (PTs1 and PTs2) participated in the reliability study (Table 2).

Forces of both lower limbs were measured with a telemetric MicroFet® 2 hand-held dynamometer (Hogan Health Industries, West Jordan, UT USA), further referred to as dynamometer. We determined segment lengths of the lower limb (fibula head—lower border of lateral malleolus) and the foot (projection of lateral malleolus on lateral border of the foot–distal metacarpal head V), and we placed the dynamometer at 75% of this segment length (S2 and S3 Figs). Each measurement consisted of one test trial, and three actual trials of a duration between three to five seconds. The resting period between each trial was at least ten seconds. Children had visual feedback during the measurements and were verbally instructed and encouraged in a standardized manner. We applied correction for gravity for the two MVICs that were influenced by gravity (knee flexion and plantar flexion), by subtracting the gravitational torque in rest position from the MVIC outcomes [30].

#### Data analysis

All parameters from the MVIC and RFD analyses were calculated with a custom-written Matlab script. First, we resampled the force data to 100 Hz and extracted the absolute maximal- and mean force (N) over three MVIC trials. Subsequently, force normalized to bodyweight (N/kg), torque (Nm), and torque normalized to bodyweight (Nm/kg) were calculated to allow comparison with previous research. Plantar flexor RFD was calculated based on: Δ force/Δ time (N/s), with a pre-fixed window size of minimally 100 ms and maximally 700 ms [31]. The onset of the measurement was determined automatically, by calculating when the force curve showed an uninterrupted increase in force based on the standard deviation of the force curve. To determine which window size would give the most reliable outcome, plantar flexor RFD was calculated for each window size from 100 ms to 700 ms with increments of 100 ms.

#### Statistical analysis

To test hypotheses 1 and 2, intra- and intertester reliability were determined for the net force and torque as well as the and plantar flexor RFD. The intra-class correlation coefficient (ICC_2,1_) based on a two-way random effect model with absolute agreement and a 95% confidence interval (CI) was calculated in SPSS (SPSS Inc., Chicago, IL) [32]. Standard error of measurement (SEM) was calculated by √MSe in which MSe is the mean squared error from the two-way Anova, representing the degree of inaccuracy between the two measurements. The minimal detectable difference (MDD) was calculated by SEM * 1.96 * √2. Both SEM and MDD were represented as a percentage of the overall mean (% SEM and % MDD respectively) to be able to compare our results with other studies. Additionally, the F-ratios were extracted from the ANOVA to determine the presence of a systematic error [32]. The F-ratio was calculated as MSm/MSr in which MSm represents systematic variance and MSr the unsystematic variance, due to unspecified, random causes. Based on the degrees of freedom in this study, a systematic error would be present when F (1,9) ≥ 5.12 [32,33].

### Part 2: Validity of the strength assessment

#### Subjects

We conducted a power analysis based on a minimal correlation coefficient (ρ_0_) of r = 0.41 and a maximal correlation coefficient (ρ_1_) of r = 0.81 (excellent), according to the classification of Altman (< 0.20 = poor; 0.21–0.40 = fair; 0.41–0.60 = moderate; 0.61–0.80 = good; 0.81–1.00 = very good) [34]. Combined with an α err prob = 0.05 and power (1-β err prob) = 0.80, this resulted in a sample size of 19 children per group (GPower 3.1.9 [35]).

A total of 23 children with CP between five and 15 years old, planned for routine clinical gait analysis, were invited to participate if they: 1) were diagnosed with bilateral or unilateral CP without signs of dyskinesia, 2) had Gross Motor Function Classification System (GMFCS) level I or II, 3) had no Botulinum Toxin-A treatment within six months prior to the assessments and 4) had no history of lower limb surgery. Twenty-three TD children of a similar age, without any neurological- or neuromuscular problems, were recruited. Three TD children participating in reliability measurements of part 1, also took part in this part of the study. General subject information of both groups is summarized in Table 3. More detailed subject information can be found in S3–S5 Tables.

**Table 3:** Subject information.

Subject information	CP	TD
	Median (25%-75%)	
Number of participants	Girls: 12; Boys: 11	Girls: 12; Boys: 11
Age (years)	9.66 (8.11–12.07)	10.04 (8.44–11.43)
Weight (kilogram)	30.5 (22.6–41.9)	31.9 (27.5–39.1)
Height (meters)	1.34 (1.21–1.48)	1.34 (1.31–1.50)
GMFCS-level	GMFCS I: 11; GMFCS II: 12	

Abbreviations in alphabetic order: GMFCS = gross motor function classification system

All children were tested at CMAL-Pellenberg and this study was approved by a local ethics committee (Commissie Medische Ethiek KU Leuven; S56041) and written informed consent was obtained from next of kin, caretakers, or guardians on behalf of the children in accordance with the Declaration of Helsinki. Children aged 12 years or older, signed the informed consent forms themselves as well.

#### Data collection

Gait kinematics and -kinetics were collected by means of 3D motion analysis. Markers were located according to the lower body Plug-in-Gait model and marker trajectories of 3D gait analyses were collected using a 10 to 15-camera VICON system (Vicon-UK, Oxford, UK), sampled at 100 Hz. Two force plates (AMTI, Watertown, MA, USA), embedded in the walkway registered force at 1500 Hz. All children walked barefoot on a 10-meter walkway at a self-selected, comfortable speed and as fast as possible without running. The latter could be considered a high demand task, potentially highlighting markers of weakness. We used Nexus software (Nexus 1.8.4. Vicon-UK, Oxford, UK) to define gait cycles and estimate the orientation of the pelvis and the joint angles of the ankle, knee and hip over the three anatomical planes, as well as the joint moments and power of the ankle, knee and hip.

MVICs were collected as described in part 1.

#### Data analysis

From the kinematic curves, we extracted: sagittal ankle angle at initial contact, sagittal knee angle at initial contact, maximal knee flexion during stance, maximal hip extension angle during mid-stance, maximal dorsiflexion angle during swing, and maximal knee flexion angle and timing of maximal knee flexion during swing. Additionally, we extracted five kinetic features from the internal net joint torques and the joint power: maximal ankle torque during loading response, maximal knee extension torque during mid-stance, maximal knee flexion torque during stance, maximal plantar flexion torque during push-off, and maximal power generation at the ankle during push-off. All parameters were extracted per gait cycle and averaged for all included gait cycles per walking speed, for each participant. All kinetic parameters were normalized to bodyweight.

For the children with CP, the gait deviations indices GDI and the GDI-kinetic [25,26] were calculated for both walking speeds for which the 23 TD children of part 2 of this study were used as the control group. Both the GDI and GDI-kinetic are measures of overall gait pathology, based on the ‘scaled distance between a pathological gait pattern and the average normal gait pattern’ [25,26]. A GDI or GDI-kinetic of 100 or higher represents a typical gait pattern. Each 10-point decrement in GDI or GDI-kinetic from 100, indicates a gait pattern that is one standard deviation away from the average TD gait pattern.

Walking speed in meters/second was extracted from the gait data and normalized to a non-dimensional value to avoid the effect of leg length [36].

From the MVICs, average net joint torque normalized to bodyweight over three trials was calculated. For the plantar flexor RFD, a window size of 300 ms was applied (RFD300), from which the absolute maximal value of the three trials was used for further analyses.

#### Statistical analysis

Since not all data were normally distributed, non-parametric tests were applied in SPSS (SPSS Inc., Chicago, IL). Group differences were tested by means of the Mann-Whitney U-test for which a Bonferroni correction was applied, resulting in a critical p-value of 0.005. The relationship between MVIC outcomes and gait parameters was checked by means of Spearman’s rank correlation coefficients for which the classification of Altman was used to interpret the results [34].

## Results

### Part 1: Reliability of the new strength assessment

The results of the reliability analyses are reported in Tables 4–6. Torque showed the highest overall reliability for all assessments with intra-tester ICCs_2,1_ between 0.681 (dorsiflexors) and 0.934 (knee flexors), and intertester ICCs_2,1_ between 0.878 (plantar flexors) and 0.947 (knee extensors). When torque was normalized to bodyweight, the ICCs_2,1_ decreased and the confidence intervals (CI) increased, resulting in ICCs_2,1_ between 0.399 (dorsiflexors) and 0.872 (knee flexors) for intra-tester measurements, and ICCs_2,1_ between 0.220 (knee extensors) and 0.647 (knee flexors) for intertester assessments. No clear difference was found between the use of the absolute maximum value and the average of three trials, but in general, the reliability was better for the averaged data (intra- and intertester), with a maximal ICC_2,1_ value of 0.951 (knee extension torque; intertester) and a minimal ICC_2,1_ value of 0.186 (knee extension normalized force; intertester).

**Table 4:** Intra-tester reliability results for the strength measurements, with the left side of the table representing the results when the absolute maximum was used and the right side of the table representing the results when an average of three trials was used. F-values are printed in bold in case of a systematic error.

		Absolute maximal value								Averaged value							
Intra-tester		F-ratio	ICC_2,1_	CI 95% *lower*	*upper*	Tester 1 *average*	Tester 2 *average*	% SEM	% MDD	F-ratio	ICC_2,1_	CI 95% *lower*	*upper*	Tester 1 *average*	Tester 2 *average*	% SEM	% MDD
**Force (N)**																	
Knee extension		0.060	0.847	0.494	0.960	237.870	235.008	11.050	30.628	0.001	0.822	0.423	0.953	223.062	223.483	11.480	31.822
Knee flexion		**8.242**	0.895	0.418	0.976	139.827	157.065	9.045	25.071	**5.942**	0.911	0.572	0.979	129.907	144.419	9.705	26.901
Dorsiflexion		1.447	0.429	-0.181	0.815	136.479	124.945	16.401	45.461	1.529	0.466	-0.134	0.830	128.949	117.758	16.406	45.475
Plantar flexion		1.098	0.690	0.182	0.911	276.963	306.767	21.786	60.387	2.722	0.715	0.234	0.919	230.362	265.125	19.018	52.715
**Normalized force (N/kg)**																	
Knee extension		0.017	0.768	0.290	0.938	7.677	7.625	11.536	31.975	0.034	0.746	0.241	0.931	7.206	7.278	12.069	33.453
Knee flexion		**8.932**	0.764	0.117	0.942	4.428	4.990	8.935	24.766	**7.540**	0.837	0.294	0.961	4.108	4.572	8.714	24.153
Dorsiflexion		1.849	0.139	-0.427	0.669	4.453	4.033	16.243	45.024	2.005	0.223	-0.350	0.713	4.208	3.799	16.169	44.817
Plantar flexion		1.030	0.620	0.059	0.888	8.928	9.797	20.442	56.662	2.537	0.669	0.160	0.904	7.461	8.493	18.171	50.367
**Torque (Nm)**																	
Knee extension		0.104	0.903	0.660	0.975	56.471	55.598	10.820	29.992	0.003	0.887	0.606	0.971	52.889	52.747	11.189	31.013
Knee flexion		**7.710**	0.934	0.593	0.985	33.646	37.639	9.023	25.011	**5.330**	0.939	0.702	0.986	31.288	34.706	10.034	27.814
Dorsiflexion		1.463	0.681	0.175	0.908	11.499	10.539	16.108	44.648	1.531	0.693	0.198	0.912	10.874	9.938	16.243	45.024
Plantar flexion		0.973	0.763	0.325	0.934	23.634	26.023	21.817	60.473	2.546	0.779	0.356	0.939	19.598	22.430	18.891	52.364
**Normalized torque (Nm/kg)**																	
Knee extension		0.045	0.806	0.385	0.948	1.802	1.782	11.300	31.321	0.008	0.775	0.305	0.940	1.689	1.697	11.812	32.740
Knee flexion		**8.706**	0.872	0.332	0.971	1.053	1.182	8.948	24.804	**6.722**	0.900	0.503	0.977	0.978	1.086	9.194	25.486
Dorsiflexion		1.896	0.399	-0.193	0.800	0.372	0.337	15.456	42.841	2.006	0.450	-0.134	0.821	0.352	0.318	16.369	45.374
Plantar flexion		0.918	0.695	0.187	0.913	0.755	0.824	20.425	56.616	2.414	0.725	0.255	0.922	0.629	0.713	17.639	48.892

Abbreviations in alphabetic order: CI = confidence interval; ICC = intra-class correlation coefficient; % MDD = minimal detectable difference as a percentage of the overall mean; N = Newton; N/kg: Newtons per kilogram bodyweight; Nm = newton meter; Nm/kg = newton meter per kilogram bodyweight; % SEM = standard error of measurement as a percentage of the overall mean.

**Table 5:** Intertester reliability results for the strength measurements, with the left side of the table representing the results when the absolute maximum was used and the right side of the table representing the results when an average of three trials was used. F-values are printed in bold in case of a systematic error.

	Absolute maximal value								Averaged value							
Intertester	F-ratio	ICC_2,1_	CI 95% *lower*	*upper*	Tester 1 *average*	Tester 2 *average*	% SEM	% MDD	F-ratio	ICC_2,1_	CI 95% *lower*	*upper*	Tester 1 *average*	Tester 2 *average*	% SEM	% MDD
**Force (N)**																
Knee extension	0.053	0.903	0.658	0.975	299.202	302.558	10.832	30.024	0.414	0.915	0.707	0.978	272.975	281.489	10.670	29.574
Knee flexion	**8.130**	0.845	0.288	0.963	149.132	177.919	13.805	38.266	**6.611**	0.860	0.388	0.966	137.667	162.349	14.309	39.663
Dorsiflexion	0.257	0.890	0.624	0.971	166.100	171.480	14.051	38.949	0.231	0.909	0.683	0.977	152.368	156.337	12.462	34.542
Plantar flexion	**19.994**	0.830	-0.005	0.965	348.567	436.446	11.196	31.034	**45.478**	0.870	-0.037	0.977	311.228	391.395	7.566	20.973
**Normalized force (N/kg)**																
Knee extension	0.008	-0.002	-0.717	0.626	8.106	8.143	11.434	31.693	0.131	0.186	-0.548	0.720	7.374	7.509	11.204	31.055
Knee flexion	**8.134**	0.547	-0.055	0.861	3.936	4.736	14.458	40.076	**7.351**	0.582	-0.021	0.877	3.600	4.289	14.452	40.058
Dorsiflexion	0.314	0.455	-0.231	0.832	4.399	4.552	13.555	37.571	0.155	0.559	-0.099	0.870	4.059	4.143	11.644	32.274
Plantar flexion	**17.095**	0.504	-0.117	0.855	9.161	11.682	13.094	36.294	**26.504**	0.543	-0.708	0.879	8.030	10.461	11.421	31.658
**Torque (Nm)**																
Knee extension	0.074	0.947	0.804	0.987	73.855	74.826	10.699	29.655	0.516	0.951	0.822	0.987	67.479	69.845	10.717	29.707
Knee flexion	**7.705**	0.892	0.433	0.975	37.075	44.147	14.026	38.877	**6.139**	0.901	0.531	0.977	34.332	40.375	14.601	40.471
Dorsiflexion	0.233	0.925	0.732	0.981	13.380	13.802	14.371	39.835	0.208	0.937	0.772	0.984	12.252	12.582	12.941	35.870
Plantar flexion	**18.173**	0.878	0.082	0.976	28.077	35.069	11.616	32.199	**44.686**	0.913	-0.008	0.985	25.221	31.453	7.355	20.387
**Normalized torque (Nm/kg)**																
Knee extension	0.007	0.220	-0.531	0.738	1.943	1.951	11.252	31.189	0.163	0.483	-0.209	0.844	1.769	1.805	10.908	30.235
Knee flexion	**8.091**	0.647	0.015	0.903	0.952	1.141	14.174	39.289	**7.107**	0.682	0.072	0.914	0.873	1.036	14.057	38.964
Dorsiflexion	0.367	0.605	-0.004	0.885	0.345	0.356	12.758	35.363	0.298	0.686	0.137	0.912	0.318	0.324	9.856	27.319
Plantar flexion	**18.155**	0.546	-0.111	0.875	0.717	0.912	12.875	35.688	**29.301**	0.604	-0.099	0.904	0.632	0.817	10.693	29.640

Abbreviations in alphabetic order: CI = confidence interval; ICC = intra-class correlation coefficient; % MDD = minimal detectable difference as a percentage of the overall mean; N = Newton; N/kg: Newtons per kilogram bodyweight; Nm = newton meter; Nm/kg = newton meter per kilogram bodyweight; % SEM = standard error of measurement as a percentage of the overall mean.

**Table 6:** Reliability outcomes from the different RFD calculations for the plantar flexors, for both intra- and intertester repeatability. The left side of the table gives the results when the absolute maximum was used, and the right side of the table are the results when an average of three trials was used. F-values are printed in bold in case of a systematic error.

	Absolute maximal value								Averaged value							
Intra- tester	F-ratio	ICC_2,1_	CI 95% *lower*	*upper*	Tester 1 *average*	Tester 2 *average*	% SEM	% MDD	F-ratio	ICC_2,1_	CI 95% *lower*	*upper*	Tester 1 *average*	Tester 2 *average*	% SEM	% MDD
**RFD (N/s)**																
RFD100	0.695	0.560	-0.052	0.869	746.625	632.15	44.543	123.467	0.292	0.636	0.043	0.896	406.529	448.258	40.423	31.822
RFD200	0.023	0.675	0.092	0.909	686.330	671.693	31.624	87.658	2.216	0.680	0.178	0.907	429.807	530.260	31.433	26.901
RFD300	0.109	0.692	0.133	0.914	583.479	608.662	28.624	79.343	3.165	0.650	0.125	0.898	386.341	490.906	29.965	45.475
RFD400	0.373	0.750	0.274	0.931	491.944	524.129	23.193	64.288	4.181	0.664	0.126	0.903	336.325	428.185	26.279	52.715
RFD500	0.458	0.752	0.283	0.932	411.351	438.618	21.210	58.792	**5.530**	0.667	0.097	0.906	285.706	366.218	23.487	33.453
RFD600	0.416	0.722	0.217	0.923	355.576	378.774	21.891	60.679	**6.696**	0.659	0.059	0.905	245.003	316.536	22.015	24.153
RFD700	0.416	0.698	0.167	0.915	308.178	328.982	22.631	62.729	**6.959**	0.627	0.022	0.894	212.017	277.660	22.727	44.817
**Intertester**																
**RFD (N/s)**																
RFD100	1.044	0.713	0.225	0.919	592.188	693.783	34.575	95.837	1.154	0.798	0.406	0.945	448.696	514.045	28.260	78.332
RFD200	0.928	0.728	0.251	0.924	717.820	808.531	27.582	76.453	1.331	0.762	0.330	0.934	563.401	645.111	26.210	72.651
RFD300	3.807	0.828	0.428	0.955	685.588	796.719	17.183	47.629	**6.906**	0.825	0.295	0.957	543.649	660.037	16.456	45.613
RFD400	**12.824**	0.836	0.116	0.964	599.439	730.474	12.305	34.108	**11.001**	0.830	0.156	0.962	490.980	601.091	13.595	37.684
RFD500	**16.701**	0.806	0.006	0.959	514.88	643.033	12.112	33.572	**6.886**	0.811	0.268	0.953	433.878	523.619	15.974	44.276
RFD600	**14.559**	0.796	0.026	0.955	451.568	564.449	13.022	36.095	**4.615**	0.787	0.309	0.944	383.234	458.565	18.630	51.639
RFD700	**14.496**	0.800	0.032	0.956	398.964	498.436	13.020	36.089	**6.543**	0.797	0.255	0.949	339.286	411.534	16.823	46.631

Abbreviations in alphabetic order: CI = confidence interval; ICC = intra-class correlation coefficient; % MDD = minimal detectable difference as a percentage of the overall mean; N/s = newtons per second; RFD = rate of force development; RFD100 = rate of force development calculation when a window of 100 milliseconds was used; RFD200 = when a window of 200 milliseconds was used; RFD300 etc; % SEM = standard error of measurement as a percentage of the overall mean.

Systematic errors were found for both absolute maximum and averaged values for the knee flexor MVICs, with F-values between 5.330 (averaged torque data) and 8.932 (maximal normalized force) for the intra-tester measurements, and F-values between 6.139 (averaged torque data) and 8.134 (maximal normalized force) for the intertester measurements. The plantar flexor MVICs also showed systematic errors for the intertester measurements with F-values between 17.095 (maximal normalized force) and 45.478 (average force). These systematic errors indicated that the second measurement outcome was always higher than the first.

For plantar flexor RFD, the use of a fixed window size of 500 ms produced the highest ICCs_2,1_ (0.752), smallest confidence intervals (CI) (0.283–0.932) and lowest % SEM (21.210) and % MDD (58.792) for the intra-tester measurements, when the absolute maximal value was used. A window size of 400 ms had the highest ICC_2,1_ value (0.836) for the intertester assessments. No clear difference was found between the use of the absolute maximal plantar flexor RFD and the average of the three trials. However, for the intra-tester assessment, the absolute maximal value gave slightly better results for the window sizes higher than 200 ms, with ICCs_2,1_ between 0.692 (RFD300) and 0.752 (RFD500). For the intertester measurements when using the absolute maximal value, ICCs_2,1_ were generally higher when the window size was higher than 300 ms with ICCs_2,1_ between 0.796 (RFD600) and 0.836 (RFD400). For the intertester measurements, systematic errors were found for all window sizes higher than 300 ms (for both absolute maximal values and averaged values), with F-values ranging between 4.615 (averaged value of RFD600) and 16.701 (maximal value of RFD500) indicating that the second assessment was significantly higher than the first.

### Part 2: Validity of the strength assessment

Group differences in gait parameters and strength data between the children with CP and the TD children are reported in Table 7. At self-selected walking speed, the children with CP showed an increased knee flexion angle at initial contact, a decreased dorsiflexion torque during loading response, a decreased hip extension angle during stance, and a lower maximal net plantar flexion torque and power generation at the ankle during push-off (all p < 0.001). The median values and interquartile ranges for the children with CP were: 25.5° (16.1) for knee angle at initial contact, -0.01 Nm/kg (0.03) for ankle torque during loading response, 4.8° (11.6) for hip angle during stance, and 1.08 Nm/kg (0.26) and 2.33 W/kg (1.13) for ankle torque and power generation at the ankle during push-off. For the TD children these values were 6.8° (5.5), -0.13 Nm/kg (0.06), -9.2° (11.1), 1.38 Nm/kg (0.22), and 4.44 W/kg (0.85). The TD children walked significantly faster than the children with CP (TD: 0.46 (0.10); CP: 0.39 (0.06); p < 0.0001).

**Table 7:** Group differences determined with a Mann Whitney U test (median, interquartile range and p-value).

	CP	TD	Mann-Whitney U
**Self-selected walking speed**	**Median (25%-75%)**		
Number of included gait cycles	4.0 (3.0–4.5)	5.0 (3.5–6.0)	
*Kinematics*			
Maximal hip extension angle during stance (deg)	4.8 (-3.2–8.4)	-9.2 (-11.7 –-0.7)	p < 0.0001*
Knee angle at initial contact (deg)	25.5 (16.4–32.5)	6.8 (3.4–8.9)	p < 0.0001*
Maximal knee flexion angle during stance (deg)	37.0 (32.3–41.3)	36.7 (1.4–39.3)	p = 0.357
Maximal knee flexion angle during swing (deg)	63.1 (48.6–66.4)	64.8 (59.9–67.6)	p = 0.017
Timing maximal knee flexion in swing (% GC)	73.2 (70.2–77.8)	70.9 (70.2–71.4)	p = 0.155
Ankle angle at initial contact (deg)	-0.1 (-2.4–3.5)	-1.4 (-4.7–0.1)	p = 0.106
Maximal dorsiflexion angle during swing (deg)	4.7 (0.2–8.1)	3.6 (1.2–5.4)	p = 0.663
*Kinetics*			
Maximal knee extension torque (Nm/kg)	0.47 (0.41–0.77)	0.62 (0.51–0.69)	p = 0.695
Maximal knee flexion torque (Nm/kg)	0.19 (-0.07–0.34)	0.20 (0.12–0.27)	p = 0.782
Ankle torque during loading response (Nm/kg)	-0.01 (-0.03 –-0.01)	-0.13 (-0.16 –-0.10)	p < 0.0001*
Maximal plantar flexion torque (Nm/kg)	1.08 (0.95–1.21)	1.38 (1.29–1.51)	p < 0.0001*
Maximal power generation at the ankle (W/kg)	2.33 (1.58–2.71)	4.44 (3.96–4.80)	p < 0.0001*
			
GDI	78.99 (73.11–87.30)		
GDI-kinetic	86.55 (77.71–93.72)		
Non-dimensional walking speed	0.39 (0.36–0.43)	0.46 (0.41–0.52)	p < 0.0001*
			
**Walking as fast as possible**			
Number of included gait cycles	3.0 (2.0–3.5)	5.0 (3.0–5.0)	
*Kinematics*			
Maximal hip extension angle during stance (deg)	6.0 (-0.5–7.3)	-10.1 (-14.6 –-2.7)	p < 0.0001*
Knee angle at initial contact (deg)	26.6 (18.1–35.1)	11.2 (7.1–14.8)	p < 0.0001*
Maximal knee flexion angle during stance (deg)	34.2 (27.5–41.2)	36.0 (33.4–39.7)	p = 0.469
Maximal knee flexion angle during swing (deg)	62.5 (50.0–66.9)	65.8 (62.0–68.473)	p = 0.071
Timing maximal knee flexion in swing (% GC)	73.2 (69.4–77.9)	69.2 (68.0–69.9)	p = 0.006
Ankle angle at initial contact (deg)	-1.4 (-3.8–2.8)	0.2 (-3.3–2.1)	p = 0.542
Maximal dorsiflexion angle during swing (deg)	5.5 (0.7–9.2)	6.6 (4.4–7.7)	p = 0.326
*Kinetics*			
Maximal knee extension torque (Nm/kg)	0.73 (0.53–1.01)	1.02 (0.63–1.15)	p = 0.326
Maximal knee flexion torque (Nm/kg)	0.50 (0.15–0.78)	0.29 (0.22–0.34)	p = 0.082
Ankle torque during loading response (Nm/kg)	-0.01 (-0.03–0.00)	-0.20 (-0.25 –-0.14)	p < 0.0001*
Maximal plantar flexion torque (Nm/kg)	1.03 (0.86–1.19)	1.49 (1.34–1.59)	p < 0.0001*
Maximal power generation at the ankle (W/kg)	2.38 (1.97–3.07)	5.52 (4.59–6.02)	p < 0.0001*
			
GDI	69.47 (62.88–79.62)		
GDI-kinetic	65.35 (61.05–71.80)		
Non-dimensional walking speed	0.55 (0.48–0.64)	0.67 (0.62–0.72)	p = 0.002*
			
**MVICS**			
Knee extension MVIC (Nm/kg)	0.48 (0.28–0.64)	1.27 (1.13–1.63)	p < 0.0001*
Knee flexion MVIC (Nm/kg)	0.43 (0.18–0.52)	0.87 (0.73–1.10)	p < 0.0001*
Dorsiflexion MVIC (Nm/kg)	0.08 (0.05–0.11)	0.27 (0.24–0.30)	p < 0.0001*
Plantar flexion MVIC (Nm/kg)	0.34 (0.22–0.38)	0.54 (0.39–0.81)	p < 0.0001*
Plantar flexion RFD (N/s)	182.40 (142.69–258.70)	661.95 (338.69–813.41)	p < 0.0001*

Abbreviations in alphabetic order: CP = cerebral palsy; deg = degrees; GDI = gait deviation index; MVIC = maximal voluntary isometric contraction; N/s = Newtons per second; Nm/kg = Newton meter per kilogram bodyweight; RFD = rate of force development; TD = typical developing; W/kg = Watts per kilogram bodyweight; % GC = percentage gait cycle. NB: All net joint torques are reported as absolute values, with exception of ankle torque during loading response (negative values represent dorsiflexion torque, positive values represent plantar flexion torque). The minimal number of included gait cycles was one in both groups (CP and TD) was one for both walking speeds.

At the higher walking speed, the same gait features were altered in CP (all p < 0.005) with median values of 26.6° (17.0) for the knee flexion angle at initial contact, -0.01 Nm/kg (0.03) for ankle torque during loading response, 6.0° (7.8) for the hip extension angle, 1.03 Nm/kg (0.33) for maximal plantar flexion torque, and 2.38 W/kg (1.09) for maximal power generation at the ankle. For the TD children these values were 11.2° (7.7), -0.20 Nm/kg (0.11), -10.1° (11.9), 1.49 Nm/kg (0.25), and 5.52 W/kg (1.43), respectively. The TD children walked significantly faster at the higher walking speed (TD: 0.67 (0.10); CP: 0.55 (0.16); p = 0.002).

For all MVIC outcomes as well as the plantar flexor RFD, the TD children showed significantly higher values than the children with CP (all p < 0.001). Median (interquartile ranges) of normalized net joint torques (Nm/kg) in children with CP were: 0.48 (0.36) for the knee extensors, 0.43 (0.33) for the knee flexors, 0.08 (0.05) for the dorsiflexors, and 0.34 (0.17) for the plantar flexors. For the TD children, these values were 1.27 (0.50), 0.87 (0.38), 0.27 (0.07), and 0.54 (0.42) respectively. The median (interquartile range) of the plantar flexor RFD (N/s) was 182.40 (116.01) for the children with CP and 661.95 (474.72) for the TD children.

The results of the correlation analysis for the children with CP are presented in Table 8. No clear intra-joint associations (for example between plantar flexion MVIC and ankle torque, or knee extension MVIC and knee angle at initial contact) were found, since correlation coefficients were always lower than 0.41. However, we did find two inter-joint relationships at self-selected walking speed: stronger plantar flexors were associated with an increased maximal knee flexion angle during swing (r = 0.61), and weaker dorsiflexors were related to a delayed timing of maximal knee flexion angle during swing (r = -0.44). At the higher walking speed this latter association disappeared, while the relationship between plantar flexion MVIC and maximal knee flexion during swing remained (r = 0.64).

**Table 8:** Spearman’s rho correlation coefficients between strength measurement outcomes and the gait parameters that differed from the TD children. Moderate or higher correlations are printed in bold.

**Self-selected walking speed**	**Knee extension MVIC (Nm/kg)**	**Knee flexion** **MVIC (Nm/kg)**	**Dorsiflexion** **MVIC (Nm/kg)**	**Plantar flexion** **MVIC (Nm/kg)**	**Plantar flexion** **RFD (N/s)**	**Non-dimensional walking speed**
Maximal hip extension angle during stance (deg)	-0.09	0.18	0.34	0.07	0.29	-0.31
Knee angle at initial contact (deg)	-0.05	0.03	0.20	-0.16	0.09	-0.42*
Maximal knee flexion angle during stance (deg)	0.33	-0.04	0.07	-.071	-.231	-.194
Maximal knee flexion angle during swing (deg)	-0.17	-0.26	-0.16	0.61**	0.37	-0.39
Timing maximal knee flexion in swing (% GC)	-0.23	-0.26	-0.44*	0.06	-0.20	-0.43*
Ankle angle at initial contact (deg)	0.00	0.22	0.11	-0.04	0.16	0.04
Maximal dorsiflexion angle during swing (deg)	0.04	0.12	0.16	-0.07	0.19	0.15
Maximal knee extension torque (Nm/kg)	-0.15	-0.02	0.07	-0.16	0.12	0.00
Maximal knee flexion torque (Nm/kg)	0.13	-0.30	-0.28	-0.16	-0.17	0.13
Ankle torque during loading response (Nm/kg)	-0.20	-0.01	0.00	0.06	-0.21	-0.20
Maximal plantar flexion torque (Nm/kg)	0.14	0.25	0.03	0.26	0.32	-0.04
Maximal power generation at the ankle (W/kg)	-0.02	-0.01	-0.30	-0.03	-0.05	0.30
GDI	0.21	-0.00	-0.05	0.12	-0.14	0.24
GDI-kinetic	0.18	0.21	-0.09	0.19	0.05	0.18
Non-dimensional walking speed	0.05	-0.12	-0.23	-0.26	-0.20	
**Walking as fast as possible**	**Knee extension MVIC (Nm/kg)**	**Knee flexion** **MVIC (Nm/kg)**	**Dorsiflexion** **MVIC (Nm/kg)**	**Plantar flexion** **MVIC (Nm/kg)**	**Plantar flexion** **RFD (N/s)**	**Non-dimensional walking speed**
Maximal hip extension angle during stance (deg)	0.04	0.23	0.40	-0.05	0.05	0.10
Knee angle at initial contact (deg)	-0.19	-0.02	0.22	-0.17	0.06	-0.38
Maximal knee flexion angle during stance (deg)	0.21	0.11	-0.01	0.00	-0.07	-0.11
Maximal knee flexion angle during swing (deg)	0.04	-0.01	0.02	0.64**	0.22	-0.03
Timing maximal knee flexion in swing (% GC)	-0.13	0.03	-0.15	0.14	-0.09	-0.23
Ankle angle at initial contact (deg)	-0.13	0.20	0.25	-0.10	0.05	0.16
Maximal dorsiflexion angle during swing (deg)	-0.08	0.12	0.26	-0.13	0.12	0.17
Maximal knee extension torque (Nm/kg)	-0.22	0.04	0.08	-0.19	-0.13	0.04
Maximal knee flexion torque (Nm/kg)	0.30	0.02	-0.36	0.02	0.08	0.31
Ankle torque during loading response (Nm/kg)	-0.13	-0.01	-0.01	-0.18	-0.10	0.02
Maximal plantar flexion torque (Nm/kg)	0.10	0.13	-0.06	0.09	0.31	0.15
Maximal power generation at the ankle (W/kg)	0.13	0.13	-0.02	-0.00	-0.02	0.50*
GDI	0.18	-0.05	-0.08	-0.00	-0.17	**0.41**
GDI-kinetic	-0.01	-0.24	0.07	-0.08	0.10	-0.49*
Non-dimensional walking speed	0.08	0.32	0.05	0.22	0.01	

Abbreviations in alphabetic order: deg = degrees; GDI = gait deviation index; MVIC = maximal voluntary isometric contraction; N/s = Newtons per second; Nm/kg = Newton meters per kilogram bodyweight; RFD = rate of force development; W/kg = Watts per kilogram bodyweight.

* p ≤ 0.05

** p ≤ 0.01

Walking speed was correlated with several altered gait features. A lower self-selected walking speed was associated with an increase in knee flexion at initial contact (r = -0.42). Children with CP who were able to walk faster at the test condition of higher walking speed, showed a higher power generation at the ankle (r = 0.50) and had a more typical kinematic gait pattern, as was represented by the fair correlation between walking speed and the GDI (r = 0.41). However, when walking at the higher walking speed, the kinetic gait pattern of the children with CP deviated further from the TD children, which was reflected by the negative correlation between walking speed and the GDI-kinetic (r = -0.49).

## Discussion

### Part 1: Reliability of the strength assessment

Our hypotheses regarding the reliability of the new strength assessment were confirmed. By fixing the children and the dynamometer, differences between intra- and intertester reliability were small when using force or torque as outcome values, suggesting that the influence of the assessor on the outcome parameters could be avoided. Normalizing the strength data to bodyweight decreased ICC_2,1_, resulted in wider confidence intervals (CIs), and increased the differences between intra- and intertester ICCs_2,1_, while % SEM and % MDD remained similar. This confirms that the ICC depends on variability of the data [32], since ICCs_2,1_ decreased when normalizing to bodyweight, even though the overall reliability remained the same. The best reliability was found for the averaged torque data, with the highest ICC_2,1_ value for knee extension strength (inter) and the lowest for dorsiflexion strength (intra) (0.951 and 0.693 resp.). Overall, our ICC_2,1_ results were in the same line as the previously reported repeatability torque data [17,20]. In our study, the ICCs_2,1_ were slightly lower for intratester reliability when compared to the results reported by Hebert et al., whereas our intertester ICCs_2,1_ were generally higher [20]. Willemse et al. showed that averaging two or three trials over one or two sessions resulted in an improved reliability when compared to using only one (maximal) value [21]. This was also the case in our study, although the differences between using the absolute maximum or over the mean of three trials were small. Unfortunately, a more detailed comparison of our reliability data to the results of previously reported studies is difficult, because the reported reliability results were often incomplete (supplementary materials S2 Table).

The systematic errors in our study were most likely the result of a learning effect during both, the intra- as well as intertester measurements. This suggests that one test trial might not have been enough practice for the TD children. However, fatiguing needs to be avoided, so the number of repetitions should be limited.

Although torque systematically showed higher ICC-values, we considered normalized torque values more suitable for future studies, due to the known strong relationship between bodyweight and strength [37]. Since % SEM and % MDD were similar for both outcome units, it may be assumed that the lower ICCs_2,1_ were mainly related to the decreased data variability due to the applied normalization. This was checked with the between subjects squared mean (as a ratio of the measurement mean) [32], which was indeed higher for the torque data than for the normalized torque values. Therefore, for future studies we recommend using the normalized torque data that are preferably averaged over multiple trials. Averaging the three trials did not only have the highest reliability outcomes, it was also considered to be more representative of the muscle activity needed during gait, since gait is a repetitive movement.

Using the fixed window size of 400 ms resulted in the highest ICC_2,1_ value (0.836) for RFD of the plantar flexors when multiple testers were used and a window size of 500 ms showed the highest intratester ICC_2,1_ value (0.752). This is contradictory to the results reported by Haff et al., who did not find an increase in ICC_2,1_ values when the window size was higher than 200 ms [31]. However, their reliability analysis was performed on college volleyball players, for whom it can be assumed that their RFD is higher than for children, possibly resulting in a smaller window size. Intertester reliability of the RFD showed systematic errors when the window size was higher than 300 ms, suggesting that the second measurement was always higher than the first. Similar to the MVICs, this might have been caused by a learning effect. Therefore, for future studies, to avoid the influence of a possible learning effect, we opted for a window size of 300 ms instead of 400 ms or 500 ms.

### Part 2: Validity of the strength measurement

We verified that the children with CP were weaker than the TD children in this study, thereby confirming our third hypothesis. The differences between children with CP and TD children regarding the MVIC outcomes are well known [13,38], and they are most likely the result of changes in motor control, and muscle- and tendon structure in CP [4,22,39,40].

Our final hypotheses were rejected due to the absence of moderate or higher (r > 0.41) intra-joint correlations between gait and muscle weakness. Muscle weakness of the plantar- and dorsiflexors was related to a decreased and delayed knee flexion angle during swing (respectively). Dorsiflexion weakness has been associated with a decreased dorsiflexion angle in swing, and in order maintain foot clearance and avoid tripping (or falling), an increased and prolonged knee flexion is often observed during swing phase [41]. This might explain the moderate correlation between dorsiflexion weakness and delayed knee flexion in swing found in the current study. However, this finding needs to be taken with some caution, since the correlation was absent at the higher walking speed, and no relationship between dorsiflexion weakness and decreased dorsiflexion angle during swing was found. Further, dorsiflexion angle in swing in CP was not significantly different from the TD children. Stronger plantar flexors are known to promote a better push off and subsequently a fluent knee flexion motion during swing [42,43]. The children with CP had indeed a lower maximal plantar flexion torque and ankle power generation during push-off, but this was not associated to weakness in the plantar flexors nor to reduced plantar flexor RFD. A possible explanation could be the contribution of the passive structures to the internal net joint torques and power generation at ankle during gait. The ability to store energy in the Achilles tendon during the second ankle rocker and release energy during push-off has been well-recognized [44]. Previous studies on the muscle and tendon structure in children with CP reported the Achilles tendon to be longer than in TD children, but with a smaller cross-sectional area [40,45]. These alterations are likely compensatory for reduced muscle compliance, resulting in a higher tendon compliance and consequently an altered ability to store and release energy [45].These findings may explain the lower maximal net joint torques during gait, and the subsequent reduced knee flexion motion in swing [4,46].

Further, during gait (passive) connective tissues are also contributing to the net joint torques and power generation via myofascial force transmission [44,47,48], while this might be less the case during MVICs. Kaya et al. found evidence that myofascial force transmission plays a role during an isometric contraction. They measured force directly at the tendon of the semitendinosus and analyzed force, joint stiffness, and range of force exertion when the semitendinosus was activated individually and when three knee flexors (gracilis, semitendinosus, and semimembranosus) were activated simultaneously [49]. They report an increase in force when the three knee flexors are activated in conjunction, but no change in stiffness or range of force exertion [49]. Their findings indicate that myofascial force transmission does have an impact on net joint torque during a MVIC, but how this relates to myofascial force transmission during a dynamic task, such as gait, needs to be explored in future studies.

Another explanation for the low correlations between MVIC outcomes and kinematic- and kinetic gait deviations could be the discrepancy in contraction types between the two measurements. During a MVIC, the muscle is contracting isometrically, whilst during gait the muscles are changing length and force. Due to the lengthening of a muscle during gait, spasticity might have an influence on gait kinematics and kinetics as well. However, muscle weakness is considered to have a more extended influence on gait kinematics and kinetics than spasticity [9,12].

Not only spasticity, but also other neuromuscular and skeletal symptoms including altered selective muscle control, muscle contractures and bony deformities are associated with gait kinematics and -kinetics in children with CP [3]. Previous research, in a study population of 200 uni- and bilateral involved children with CP, indicated only poor to moderate correlations between pre-determined gait features, and passive range of motion (r ≤ 0.51), spasticity (r ≤ 0.50) and selective motor control (r ≤ 0.50) [9]. Within the group of children with CP in this study, the correlations between clinical symptoms and gait features at self-selected walking ranged between poor to good. For passive range of motion, the highest correlation was found between passive hip extension mobility and knee angle at initial contact (r = -0.61). Increased spasticity of the knee flexors was associated with increased knee flexion angle at initial contact (r = 0.73), and decreased selective control of the plantar flexors with decreased power generation at push-off (r = 0.75). Moreover, the clinical symptoms are inter-correlated [50]. A more involved child frequently has a higher level of spasticity, lower selective muscle control, and more muscle weakness, than a less involved child [50,51]. Our study sample size did not have sufficient power for detailed analyses of the interaction between various clinical symptoms and their combined relation to the gait deviations in CP.

Several studies used musculoskeletal models to analyze the effect of muscle weakness on gait kinematics and kinetics [5,52]. Van der Krogt et al. found that, in case of weakness of the hip abductors, hip flexors, and plantar flexors, normal gait is hard to maintain [5]. Similar results are reported by Steele et al., who determined that weakness of the hip abductors and the plantar flexors are contributing to crouch gait in children with CP [52]. The use of (subject-specific) musculoskeletal models in analyzing the underlying neuromuscular deficits contributing to muscle weakness and their interaction with gait, could further enhance our understanding into the relationship between muscle weakness and gait deviations in children with CP.

We measured MVICs in a standardized test position, while Ateş et al. and Yucesoy et al. determined that the optimal muscle length to deliver force differs per individual in CP [53–55]. They intraoperatively assessed maximal isometric force of three different spastic knee flexors (gracilis, semitendinosus, and semimembranosus) in children with CP. Force was measured directly at the tendon and at different joint angles [53–55]. Their results indicate inter-subject variability regarding the optimal muscle length for generating isometric force in the three knee flexors [53–55]. This is in line with the wide range of joint torques we found for the knee flexion MVICs (minimal torque: 0.04; maximal torque 0.98 (Nm/kg)). Additionally, they determined that the angle at which these three knee flexors are able to generate their peak force differs per muscle, thereby allowing for a wider range of motion at which knee flexion torque can be generated [55]. This is very useful during dynamic tasks such as gait, and indicates that the maximal knee flexion torque measured with a MVIC could be an underestimation. These findings imply that there might not be one optimal test position to assess muscle weakness, but that test positions should ideally be muscle and subject specific.

Finally, walking speed has a big influence of gait kinematics and kinetics [56]. The TD children in this study walked faster than the children with CP at both walking speeds, which appears to have been the main reason behind the differences in kinematic- and kinetic parameters [56]. This was confirmed by the moderate correlations between self-selected walking speed, and the knee angle at initial contact (r = -0.42), and between the higher walking speed and power generation at the ankle (r = 0.50), the GDI (r = 0.41) and the GDI-kinetic (r = -0.49). The latter negative correlation between the high walking speed and the GDI-kinetic suggests that the children with CP used an altered gait strategy to increase their walking speed when compared to the TD children in this study. Riad et al. found a shift in power generation from the ankle to the hip during gait in children with CP, indicating an alternate strategy from ankle push-off to a hip pull-off, at pre-swing [57]. Analysis of the power generation at the hip of the participants in this study revealed that the children with CP were indeed prone to increase the hip-pull off, opposed to an ankle push-off, to increase walking speed.

One of the limitations of part 1 of this study was that both sides (left and right) were included in the reliability analysis. Also, the reliability study was only performed on TD children and should be extended with data from children with CP in future studies.

Further, during the weakness assessment, we selected a test position with joint angles that were representative for the averaged joint angles during gait found in children with CP, not TD children. We verified that the averaged joint angles for the TD children participating in part 2 were similar as the angles extracted from our retrospective dataset of children with CP (hip: 18.4^0^ ± 6.5^0^; knee: 25.5 ± 3.9^0^; ankle: 2.7 ± 4.7^0^) [29].

Additionally, we combined both unilateral and bilateral CP even though the natural history of certain clinical symptoms, such as level of spasticity and muscle weakness, is expected to be different between both patient groups. However, a separate data analysis revealed that there were no significant differences (Mann-Whitney U test) for passive range of motion, muscle strength, GDI or GDI-kinetic between children that were bilaterally or unilaterally involved.

## Conclusion

The reliability of our new weakness assessment was found to be good. However, based on the weak intra-joint correlations between MVIC-outcomes and the altered kinematic- and kinetic gait parameters, validity is considered to be poor. We did find a strong association between weakness of the dorsiflexors and plantar flexors with delayed and decreased peak knee angle during swing. These inter-joint associations indicate that the relationship between muscle weakness and altered gait is complex. Also, the interdependency of muscle weakness with other neuromuscular symptoms, such as decreased passive range of motion, reduced selective motor control, and spasticity, should be taken into consideration in future studies.

## Supporting information

S1 Fig Custom made chair used for the MVIC measurements. Hip and knee joints are placed in 30^0^ flexion. The ankle is placed in neutral position.
(TIF)

S2 Fig Custom made chair used for the MVIC measurements, with fixed location of the hand-held dynamometer during knee-extension MVIC. The black + red lines represent the segment length (fibula head—lower border of lateral malleolus). The black line indicates the moment arm (75% of the segment length).
(TIF)

S3 Fig Custom made chair used for the MVIC measurements, with fixed location of the hand-held dynamometer during dorsiflexion MVIC. The red line represents the segment length (projection of lateral malleolus on lateral border of the foot–distal metacarpal head V). The black line indicates the moment arm (75% of the segment length).
(TIF)

S1 Table Overview of characteristics of the studies analyzing the relationship between muscle weakness and gait in children with CP. Abbreviations in alphabetic order: allF = all strength measurements; aggF = aggregated strength measurements; avF = average of the strength measurements; DF = dorsiflexion; Habd = hip abduction; Hadd = hip adduction; HE = hip extension; HF = hip flexion; HHD = hand-held dynamometry; ID = isokinetic device; KE = knee extension; KF = knee flexion; MMT = manual muscle testing; MVCC = maximal voluntary concentric contraction; MVIC = maximal voluntary isometric contraction; NPF = plantar flexion; v1 = self-selected walking speed; v2 = faster walking speed;? = unclear which values, units, protocol or calculations have been used.
^1^ Only fair (r ≥ 0.21) or higher correlations are listed. ^2^ Only the differences between group 1 and 2 are reported, indicating differences between the stronger vs the weaker children with CP. ^3^ Median (min-max), instead of mean ± SD.
An increase in a value is indicated with a ↑ and a decrease with a ↓. For instance, when looking at pelvic range of motion, Ross & Engsberg found that when the aggregated strength values of the tested muscles (aggF) decreased, pelvic ROM increased (↓ aggF ↑).
For each study, only significant results are reported, unless the same parameter was also tested in another study in which they found significant results, such as cadence e.g.
(DOCX)

S2 Table Results of intra- or intertester reliability studies on the use of the HHD in CP and/or TD children between 5–15 years of age. Only studies employing the make test and using ICCs as reliability metrics are summarized. When left and right side were tested separately, data was averaged for clarity. In case of multiple test protocols or reliability assessments, only the results of the underlined tests and the results of the highest reported ICCs (indicated with a *) have been reported. If possible, parameters were calculated when missing from the paper.
Abbreviations in alphabetic order: Av: average; CI = confidence interval; CP = cerebral palsy; DF = dorsiflexion; Dx = diagnosis; Habd = hip abduction; HE = hip extension; HF = hip flexion; ICC = intra-class correlation coefficient; HHD = Hand-held dynamometer, KE = knee extension; KF = knee flexion; Kg = kilogram; lbs = pounds; % MDD = minimal detectable difference as a percentage of the overall mean; N = Newtons; PF = plantar flexion; % SEM = standard error of measurement as a percentage of the overall mean; TD = typical developing;? = unclear which values, units, protocol or calculations have been used.
^1^ Left and right side were analyzed separately, but the values were averaged for clarity; ^2^ Significant differences between the two sessions for the weaker leg; ^3^ Significant differences between the two sessions for the stronger leg.
(DOCX)

S3 Table Detailed subject and measurement information of the typical developing (TD) children participating in part 2 of this study. (DOCX)

S4 Table Detailed subject specifics regarding gait parameters for the children with CP participating in part 2 of this study. Abbreviations in alphabetic order: CP = cerebral palsy; GDI = gait deviation index; GMFCS = gross motor function classification scale. NB: Gait joint pattern descriptions can be found in S6 Table.
(DOCX)

S5 Table Detailed subject characteristic of the children with CP regarding clinical outcome measures. Passive range of motion for dorsiflexion was performed with extended knee. Spasticity is graded with the Modified Ashworth scale. For the plantar flexors, this was done with flexed- (90^0^) and extended knee (0^0^).
Abbreviations in alphabetic order: DF = dorsiflexion; HE = hip extension; HF = hip flexion; KE = knee extension; KF = knee flexion; PF = plantar flexion; PROM = passive range of motion; SPAS = spasticity.
(DOCX)

S6 Table Description of gait classification patterns. Classifications are derived from the paper of Nieuwenhuys et al: Identification of joint pattern during gait in children with cerebral palsy: a Delphi consensus study, Developmental Medicine and Child Neurology 2016, 58: 306–313 [58].
(DOCX)
