# Supplementary figures and images for: A new strength assessment to evaluate the association between muscle weakness and gait pathology in children with cerebral palsy
Source: PLoS One. 2018 Jan 11;13(1):e0191097. doi: 10.1371/journal.pone.0191097 (PMC5764363; doi:10.1371/journal.pone.0191097)

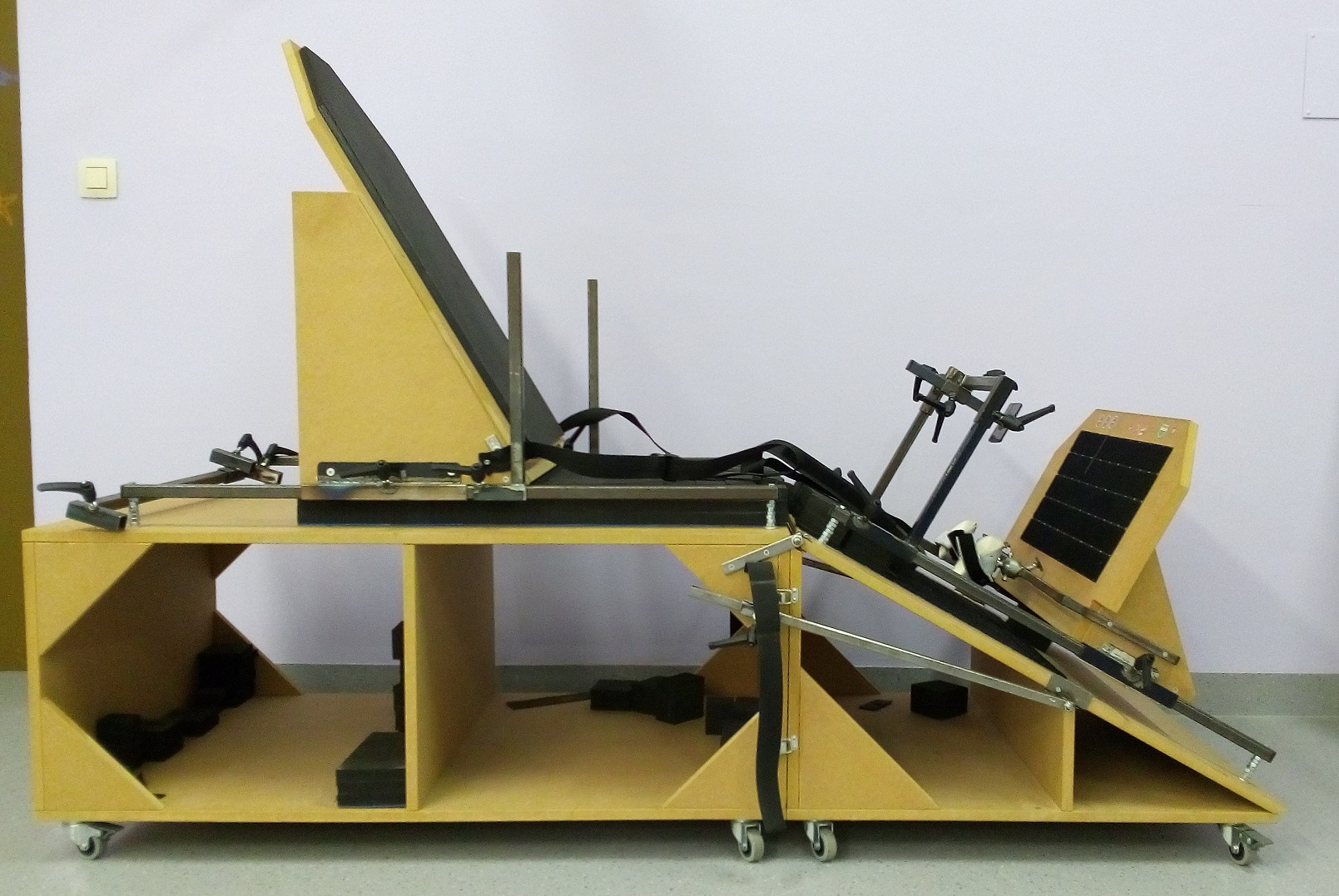

Supplement: S1 Fig — Hip and knee joints are placed in 300 flexion. The ankle is placed in neutral position. (TIF) [file pone.0191097.s001.tif]

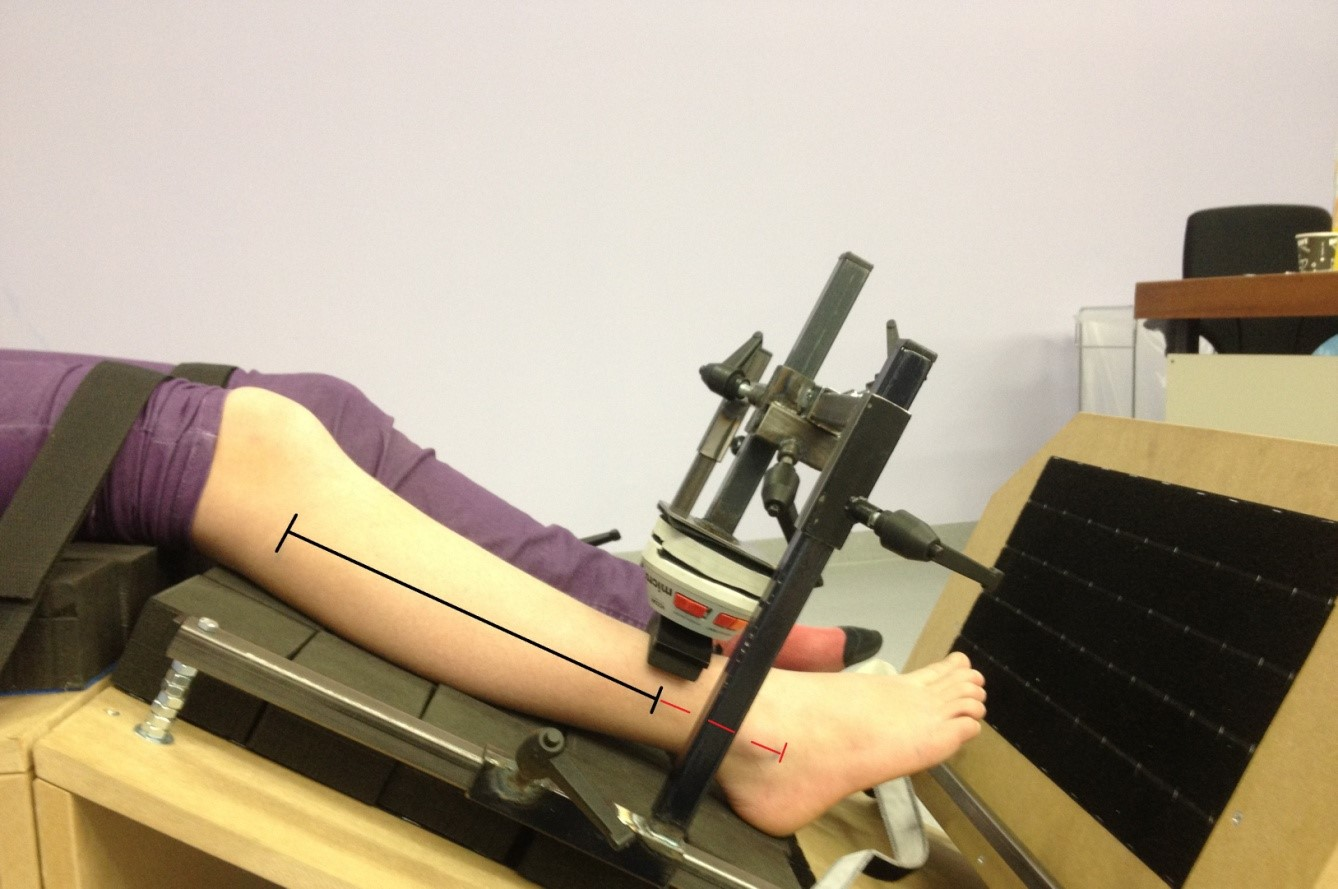

Supplement: S2 Fig — The black + red lines represent the segment length (fibula head—lower border of lateral malleolus). The black line indicates the moment arm (75% of the segment length). (TIF) [file pone.0191097.s002.tif]

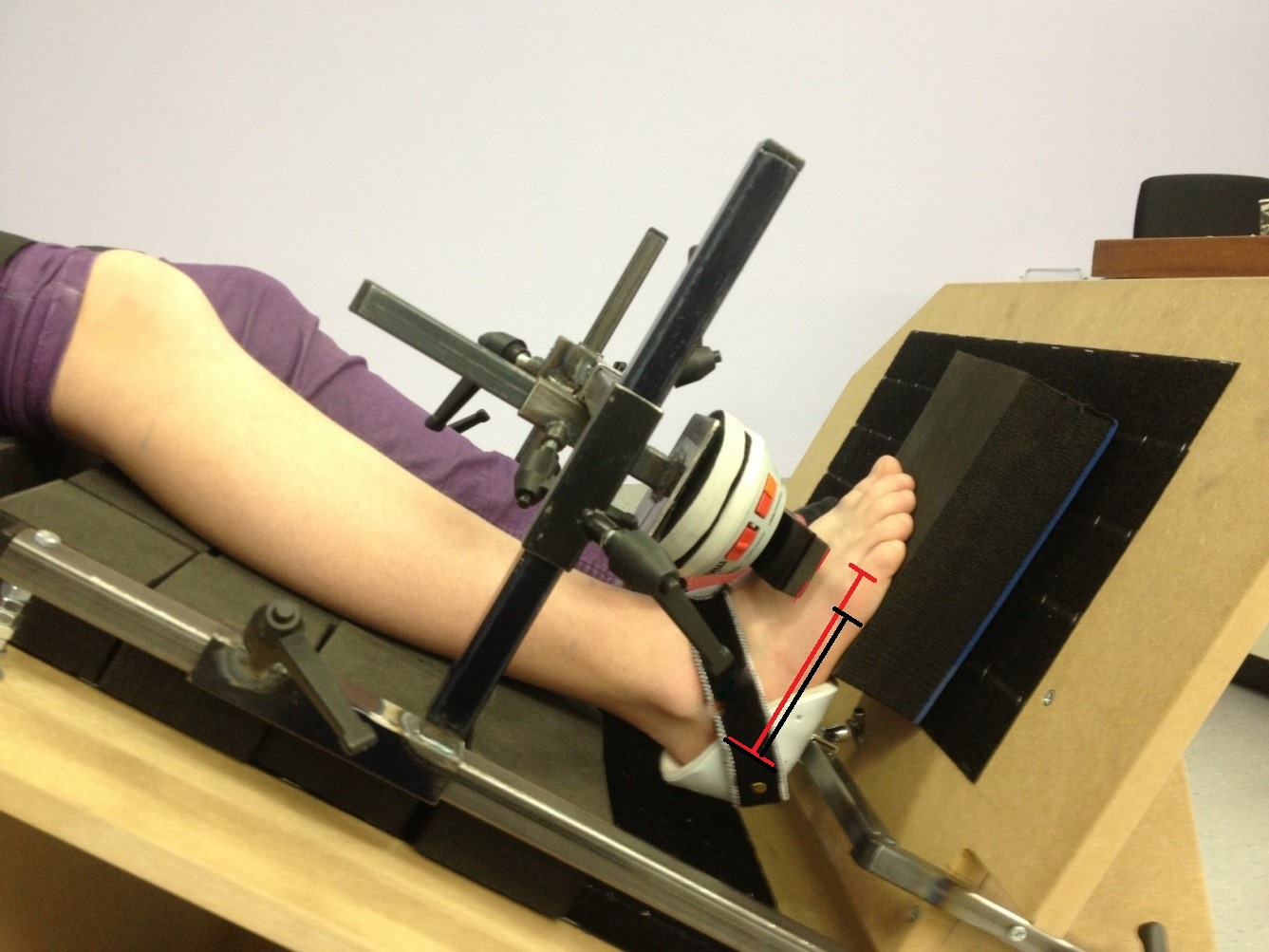

Supplement: S3 Fig — The red line represents the segment length (projection of lateral malleolus on lateral border of the foot–distal metacarpal head V). The black line indicates the moment arm (75% of the segment length). (TIF) [file pone.0191097.s003.tif]
