# Supplementary material for: A new strength assessment to evaluate the association between muscle weakness and gait pathology in children with cerebral palsy
Source: PLoS One. 2018 Jan 11;13(1):e0191097. doi: 10.1371/journal.pone.0191097 (PMC5764363; doi:10.1371/journal.pone.0191097)
Supplement: S1 Table — Abbreviations in alphabetic order: allF = all strength measurements; aggF = aggregated strength measurements; avF = average of the strength measurements; DF = dorsiflexion; Habd = hip abduction; Hadd = hip adduction; HE = hip extension; HF = hip flexion; HHD = hand-held dynamometry; ID = isokinetic device; KE = knee extension; KF = knee flexion; MMT = manual muscle testing; MVCC = maximal voluntary concentric contraction; MVIC = maximal voluntary isometric contraction; NPF = plantar flexion; v1 = self-selected walking speed; v2 = faster walking speed;? = unclear which values, units, protocol or calculations have been used. 1 Only fair (r ≥ 0.21) or higher correlations are listed. 2 Only the differences between group 1 and 2 are reported, indicating differences between the stronger vs the weaker children with CP. 3 Median (min-max), instead of mean ± SD. An increase in a value is indicated with a ↑ and a decrease with a ↓. For instance, when looking at pelvic range of motion, Ross & Engsberg found that when the aggregated strength values of the tested muscles (aggF) decreased, pelvic ROM increased (↓ aggF ↑). For each study, only significant results are reported, unless the same parameter was also tested in another study in which they found significant results, such as cadence e.g. (DOCX) [file pone.0191097.s004.docx]

|  | Damiano et al.  1995 [6] | Damiano et al 1998 [7] | Damiano et al  2010 [8] | Desloovere et al 2006^1^ [9] | Meyns et al  2016^2^ [10] | Engsberg et al  2006 [11] | Ross & Engsberg 2007 [12] | Eek et al  2011 [13] | Lee et al  2008 [14] | Shin et al  2016 [15] |
| --- | --- | --- | --- | --- | --- | --- | --- | --- | --- | --- |
| Study population |  |  |  |  |  |  |  |  |  |  |
| Number of children with CP | 14 | 11 | 8 | 200 | 30 | 12 | 97 | 20 | 16 | 24 |
| Diagnosis specifications | Diplegic | 6 Diplegic  5 Hemiplegic | ? | 112 Diplegic  88 Hemiplegic | 18 Diplegic  12 Hemiplegic | Diplegic | Diplegic | Diplegic | 9 Diplegic  8 Hemiplegic | 18 Diplegic  6 Hemiplegic |
| Age (mean ± standard deviation) | 9.1 ± 2.5 | 8.2 ± 2.3 | 7.8 ± 2.6 | 8.1 ± 2.4 | 8.5 ± 2.3 | 9.9 ± 3.4 | 9.1 ± 4.8 | 12.9 (9.4-15.3) ^3^ | 6.3 ± 2.5 | 10.0 ± 5.2 |
| Stationary measurement |  |  |  |  |  |  |  |  |  |  |
| Test condition | Indirect | Indirect | Indirect | Direct | Indirect | Indirect | Direct | Direct | Indirect | Direct |
| Measurement method | HHD | HHD | ID | MMT | MMT | ID | ID | HHD | MMT | HHD |
| Measurement protocol | 6 weeks  3 sessions/week | 6 weeks  3 sessions/week | 8 weeks  3 sessions/week |  |  | 12 weeks  3 sessions/week |  |  | 5 weeks  3 sessions/week |  |
| Extracted parameters | Max MVIC | Max MVIC | Max MVCC | Max MVIC | Max MVIC | Max MVCC + MVEC | Max MVCC | Max MVIC | Max MVIC | Max MVIC |
| Outcome units | N | N as % TD | Nm/kg | 10-point scale | Group 1: ≥ 4  Group 2: 2.5-4 | Nm/kg | Nm/kg | Nm/kg | 5-point scale? | N/kg |
| Description of test position | Yes | Yes | Yes | Not mentioned | Not mentioned | Yes | Yes | Yes | Not mentioned | Yes |
| Gravity correction applied | Not mentioned for non-neutral | Not mentioned for non-neutral | Not mentioned | Not mentioned | Not mentioned | Not mentioned | Not mentioned | Not mentioned for non-neutral | Not mentioned | Gravity neutral positions |
| *Muscles tested* | | | | | | | | | | |
| *Hip extensors* |  | HE | HE | HE |  |  |  | HE | HE | HE |
| *Hip flexors* |  | HF |  |  |  |  |  | HF | HF | HF |
| *Hip abductors* |  | Habd |  | Habd |  |  | Habd | Habd | Habd |  |
| *Hip adductors* |  | Hadd |  |  |  |  | Hadd | Hadd | Hadd |  |
| *Knee extensors in 90^0^ knee flexion* | KE | KE | KE | KE | KE |  | KE | KE | KE | KE |
| *Knee flexors in 90^0^*  *knee flexion* | KF | KF |  | KF | KF |  | KF | KF | KF | KF |
| *Dorsiflexors in* 0^0^*, 90^0^ or other knee flexion position* |  | DF 90^0^ |  | DF 0^0^-90^0^ | PF | DF other | DF other | DF 90^0^ |  |  |
| *Plantar flexors* |  | PF |  | PF |  | PF | PF | PF |  |  |
| Functional measurement | | | | | | | | | | |
| Measurement method | 3D-gait analysis | 3D-gait analysis | 3D-gait analysis | 3D-gait analysis | 3D-gait analysis | 3D-gait analysis | 3D-gait analysis | 3D-gait analysis | 3D-gait analysis | 3D-gait analysis |
| Measurement protocol | v1 & v2 | v1 & v2 | v1 & v2 | v1 | v1 & v1+weight | v1 | v1 | v1 | v1 | v1 |
| *Spatiotemporal parameters* | | | | | | | | | | |
| *Cadence* | Not significant  at v1+v2 | ↑ avF ↑  at v1+v2 | Not significant.  at v1+v2 | Not significant |  | Not significant | ↑ PF ↑ |  | Not significant | Not significant |
| *Walking speed* | Not significant  at v1+v2 | ↑ avF ↑  at v1+v2 | N.S.  at v1+v2 | ↑ allF ↑  except DF | Not significant | Not significant | ↑ Habd, PF ↑ |  | ↑ allF or HE ↑? | Not significant |
| *Step length* |  |  |  | ↑ allF ↑ ­  except DF + PF | Not significant |  |  |  |  |  |
| *Stride length* | ↑ KE ↑  at v1+v2 | Not significant  at v1+v2 | Not significant  at v1+v2 |  | Not significant | Not significant | ↑ DF, KE, Habd↑ |  | ↑ allF or HE ↑? | Not significant |
| *Double support*  *time* |  | ↓ avF ↑  at v2 |  |  | Not significant |  |  |  | ↑ allF or HE ↓? |  |
| *Kinematic parameters* | | | | | | | | | | |
| *Pelvic range of motion (deg)* |  |  |  | ↓ allF ↑  Except HE |  |  | ↓ aggF ↑ |  |  |  |
| *Mean pelvic tilt (deg)* |  |  |  | Not significant |  |  |  |  |  | ↓ HE, KE ↑ |
| *Hip angle in*  *terminal stance (deg)* |  |  |  | ↓ KE, KF, PF ↑ |  |  |  |  |  |  |
| *Maximal hip flexion (deg)* |  |  |  | Not significant | Not significant |  |  |  | ↑ allF or HE ↑? | ↓ HE ↑ |
| *Hip range of motion during stance (deg)* | Not significant |  |  | ↑ allF ↑  Except DF | Not significant |  | Not significant |  |  |  |
| *Knee flexion during loading response (deg)* | ↓ KE ↑  at v1 |  |  | ↑ allF ↑ |  |  | ↓ aggF ↑ |  |  | ↑ KE ↑ |
| *Minimal knee flexion angle (deg)* |  |  |  |  |  | ↓ allF ↑ |  |  |  | ↑ KE ↑ |
| *Timing maximal knee flexion angle in swing (% GC)* |  |  |  | ↓ allF ↑ ­  except HE + KE |  |  |  |  |  |  |
| *Maximal knee flexion angle in swing (deg)* |  |  |  | ↑ Habd ↑ |  |  |  |  |  | ↑ KE ↑ |
| *Maximal angular velocity at the knee around toe-off* |  |  |  | ↑ Habd,KE,PF ↑ |  |  |  |  |  |  |
| *Knee flexion range of motion (deg)* |  |  |  |  |  |  | ↑ aggF ↑ |  |  |  |
| *Ankle range of motion around push-off (deg)* |  |  |  | ↑HE,Habd,  KF ↑ ­ |  |  |  |  |  |  |
| *Maximal dorsiflexion angle during stance* |  |  |  | ↑ DF ↑ ­ |  | Not significant |  |  |  |  |
| *Maximal angular velocity at the ankle around toe-off* |  |  |  | ↓ PF ↑ ­ |  |  |  |  |  |  |
| *Dorsiflexion angle at initial contact* |  |  |  |  |  | Not significant | ↑ aggF ↑ |  |  |  |
| *Kinetic parameters* | | | | | | | | | | |
| *Maximal net internal hip flexion torque* |  |  |  |  |  |  |  | Not significant | ↑ allF or HE ↑? |  |
| *Maximal net internal knee extension torque* |  |  |  | Not significant |  |  |  | Not significant |  | ↑ KE ↑ |
| *Maximal net internal plantar flexion torque* |  |  |  | ↑ allF ↑  Except KF + DF90 |  |  |  | ↑ allF ↑­  except HE + HF |  |  |
| *Maximal power generation at the hip at H3* |  |  |  | ↑ Habd ↑ | Not significant |  |  | ↑ KE ↑ |  |  |
| *Maximal power absorption at the knee at K4* |  |  |  | Not significant |  |  |  |  |  | ↑ allF ↑ |
| *Maximal power generation at the ankle during A2* |  |  |  | ↑ allF ↑ |  |  |  | ↑ allF ↑ |  |  |
