# Supplementary material for: A new strength assessment to evaluate the association between muscle weakness and gait pathology in children with cerebral palsy
Source: PLoS One. 2018 Jan 11;13(1):e0191097. doi: 10.1371/journal.pone.0191097 (PMC5764363; doi:10.1371/journal.pone.0191097)
Supplement: S2 Table — Only studies employing the make test and using ICCs as reliability metrics are summarized. When left and right side were tested separately, data was averaged for clarity. In case of multiple test protocols or reliability assessments, only the results of the underlined tests and the results of the highest reported ICCs (indicated with a *) have been reported. If possible, parameters were calculated when missing from the paper. Abbreviations in alphabetic order: Av: average; CI = confidence interval; CP = cerebral palsy; DF = dorsiflexion; Dx = diagnosis; Habd = hip abduction; HE = hip extension; HF = hip flexion; ICC = intra-class correlation coefficient; HHD = Hand-held dynamometer, KE = knee extension; KF = knee flexion; Kg = kilogram; lbs = pounds; % MDD = minimal detectable difference as a percentage of the overall mean; N = Newtons; PF = plantar flexion; % SEM = standard error of measurement as a percentage of the overall mean; TD = typical developing;? = unclear which values, units, protocol or calculations have been used. 1 Left and right side were analyzed separately, but the values were averaged for clarity; 2 Significant differences between the two sessions for the weaker leg; 3 Significant differences between the two sessions for the stronger leg. (DOCX) [file pone.0191097.s005.docx]

|  | Study population | Test  protocol | Test position described | Data analysis used | Reliability  assessment | Muscles tested | ICC results | 95% CI ICC  Lower - higher | % SEM | % MDD | F- or p-value anova |
| --- | --- | --- | --- | --- | --- | --- | --- | --- | --- | --- | --- |
| Berry  et al 2004 [16] | N: 15 CP  Dx: 12 Diplegic & 3 Quadriplegic  Age: 11.7 ± 3.6 | Make  3 sec  3 trials  2 weeks between sessions | Yes  Gravity correction: gravity neutral positions | Max - Av  Force (N) or normalized force (N/lbs) | Intra  ICC_3,1_  Within  Between | Habd  KE  KF | Max - Av  0.95 - 0.95  0.95 - 0.96  0.79 - 0.78 | Not mentioned | Measured but unclear if max or average values were used | Could not be calculated | Not mentioned |
| Eek  et al 2006 [17] | N: 149 TD  Age: not mentioned only a range is given (5-15 yrs) | Make  5 sec  3 trials  0 days? | Yes  Gravity correction: not mentioned for non-neutral gravity positions | Maximal torque (Nm) | Inter  ICC_2,_ type? | HF  KE  KF | Only a range is given for all three  0.93-0.97 | Not mentioned | Not mentioned | Not mentioned | No significant differences between the two measurements |
| Crompton et al 2007 [18] | N: 23 CP  Dx: Diplegic  Age: 9.6 ± 2.8 | Make  3-5 sec  2 trials  1 week between sessions | Yes  Gravity correction: not mentioned for non-neutral gravity positions | Maximal Normalized force (N/kg) | Intra  ICC_1,1_  Within  Between | HF  HE supine  HE prone  KF  KE 90^0^  KE 20^0^  PF  DF | Not fixed- Fixed1  0.80  0.81^*^- 0.74  0.33  0.77  0.86^*^- 0.80  0.56  0.68  0.65 - 0.77^*^ | 0.60 - 0.92  0.60 - 0.91  -0.11 - 0.66  0.53 - 0.90  0.70 - 0.94  0.20 - 0.79  0.37 - 0.86  0.50 - 0.90 | Not fixed- Fixed1  16.8  20.4 - 55.9  48.1  24.0  16.1 - 19.6  42.4  31.0  30.4 - 24.4 | Not fixed- Fixed1  35.7  56.5 - 154.9  133.32  66.5  44.6 - 54.3  117.5  85.9  84.3 - 67.6 | Not fixed- Fixed1  p<0.052- p<0.05  p < 0.05  p < 0.013  p < 0.05 |
| Verschuren  et al 2008 [19] | N: 25 CP  Dx: 12 Diplegic, 13 Hemiplegic  Age: 10.6 ± 2.7 | Make vs Break vs functional  Duration?  3 trials  0 days between sessions | Yes  Gravity correction: gravity neutral positions | Maximal?  Force (N) | Inter  ICC type? | HE  Habd  KE  KF  PF | 0.75  0.69  0.70  0.57  0.22 | Not mentioned | 16.0  22.8  21.5  25.0  34.6 | 44.3  63.2  59.6  69.3  95.9 | Not mentioned |
| Hebert  et al 2011 [20] | N: 74 TD  Age: 10.7 ± 3.9 | Make  10 sec  2 trials  5-14 days between sessions | Yes  Gravity correction: gravity neutral positions | Average of 2 trials  Torque (Nm) | Intra - inter  ICC_1,1_ - ICC_2,1_ | HF  HE  Habd  KE  KF  DF  PF | Intra - Inter  0.98^*^- 0.95  0.84^*^ - 0.75  0.81 - 0.88^*^  0.90^*^ - 0.84  0.95^*^- 0.93  0.79^*^- 0.67  0.92^*^- 0.87 | 0.92 - 0.99  0.45 - 0.96  0.59 - 0.97  0.65 - 0.97  0.81 - 0.99  0.36 - 0.94  0.71 - 0.98 | Calculated, but average torque value was not reported as a number | Could not be calculated | Not mentioned |
| Willemse  et al 2013 [21] | N: 14 CP  Dx: 8 Diplegic, 6 Hemiplegic  A:10.2 ± ? | Make  3-5 sec  3 trials  2-5 days between sessions | Yes  Gravity correction: not mentioned for non-neutral gravity positions | Maximal value - Average of 2 trials - Average of 3 trials  Normalized force (N/kg) | Intra  ICC type?  Within  Between | HF  Habd  KE  KF  PF | 1-2-3  0.82 -0.86-0.87^*^  0.88-0.89-0.90^*^  0.88-0.89-0.90^*^  0.95-0.96-0.97^*^  0.87-0.88-0.98^*^ | 0.81 - 0.91  0.85 - 0.93  0.85 - 0.93  0.95 - 0.98  0.84 - 0.93 | 1-2-3  10.1-8.9-8.5  13.3-12.2-11.8  9.0-8.4-8.1  8.6-7.4-7.0  13.6-12.5-12.2 | 1-2-3  28.0-24.7-23.5  36.8-33.7-32.6  24.9-23.2-22.6  23.8-20.6-19.4  37.7-34.8-33.8 | Not mentioned |
