# Supplementary material for: A new strength assessment to evaluate the association between muscle weakness and gait pathology in children with cerebral palsy
Source: PLoS One. 2018 Jan 11;13(1):e0191097. doi: 10.1371/journal.pone.0191097 (PMC5764363; doi:10.1371/journal.pone.0191097)
Supplement: S3 Table — (DOCX) [file pone.0191097.s006.docx]

| TD | Gender | Age  *years* | Weight  *kilogram* | Height  *meters* |
| --- | --- | --- | --- | --- |
| TD21 | Girl | 13.70 | 71.6 | 1.67 |
| TD22 | Girl | 13.05 | 41.9 | 1.58 |
| TD23 | Boy | 13.71 | 38.2 | 1.53 |
| TD24 | Girl | 10.92 | 57.0 | 1.30 |
| TD25 | Girl | 10.65 | 33.2 | 1.47 |
| TD18 | Girl | 10.84 | 37.0 | 1.31 |
| TD26 | Girl | 10.41 | 30.3 | 1.40 |
| TD27 | Boy | 10.14 | 34.4 | 1.45 |
| TD28 | Girl | 10.03 | 22.6 | 1.31 |
| TD29 | Girl | 10.04 | 37.8 | 1.41 |
| TD30 | Boy | 8.61 | 31.9 | 1.34 |
| TD31 | Boy | 8.55 | 27.5 | 1.34 |
| TD11 | Boy | 7.93 | 29.9 | 1.33 |
| TD32 | Girl | 9.17 | 30.3 | 1.36 |
| TD33 | Boy | 7.30 | 23.0 | 1.27 |
| TD34 | Boy | 11.93 | 39.9 | 1.53 |
| TD35 | Boy | 11.95 | 46.4 | 1.59 |
| TD36 | Boy | 15.44 | 50.4 | 1.66 |
| TD37 | Girl | 7.80 | 27.4 | 1.26 |
| TD38 | Boy | 6.29 | 21.9 | 1.18 |
| TD5 | Girl | 8.29 | 29.6 | 1.29 |
| TD1 | Girl | 8.70 | 27.3 | 1.31 |
| TD2 | Boy | 8.32 | 25.6 | 1.32 |
