# Supplementary material for: A new strength assessment to evaluate the association between muscle weakness and gait pathology in children with cerebral palsy
Source: PLoS One. 2018 Jan 11;13(1):e0191097. doi: 10.1371/journal.pone.0191097 (PMC5764363; doi:10.1371/journal.pone.0191097)
Supplement: S4 Table — Abbreviations in alphabetic order: CP = cerebral palsy; GDI = gait deviation index; GMFCS = gross motor function classification scale. NB: Gait joint pattern descriptions can be found in S6 Table. (DOCX) [file pone.0191097.s007.docx]

| CP | Specific diagnosis | GMFCS | Side included | GDI  Self-selected | GDI  As fast as possible | GDI-kinetic  Self-selected | GDI-kinetic  As fast as possible | Knee in stance  Self-selected | Knee in swing  Self-selected | Ankle in stance  Self-selected | Ankle in swing  Self-selected |
| --- | --- | --- | --- | --- | --- | --- | --- | --- | --- | --- | --- |
| CP1 | Diplegic | 1 | Left | 89.7 | 78.7 | 111.9 | 65.6 | KStS6 | KSwS0 | AStS0 | ASwS0 |
| CP2 | Hemiplegic right | 1 | Right | 89.9 | 93.4 | 78.7 | 67.7 | KStS0 | KSwS4 | AStS0 | ASwS0 |
| CP3 | Hemiplegic left | 2 | Left | 80.9 | 70.0 | 89.3 | 77.1 | KStS5 | KSwS0 | AStS3 | ASwS2 |
| CP4 | Diplegic | 1 | Right | 94.2 | 91.3 | 80.5 | 70.6 | KStS6 | KSwS3 | AStS2 | ASwS3 |
| CP5 | Diplegic | 1 | Right | 110.4 | 98.8 | 108.0 | 58.5 | KStS0 | KSwS0 | AStS0 | ASwS0 |
| CP6 | Hemiplegic right | 1 | Right | 86.3 | 82.4 | 107.4 | 73.0 | KStS6 | KSwS1 | AStS0 | ASwS0 |
| CP7 | Diplegic | 2 | Right | 80.2 | 80.6 | 86.6 | 78.9 | KStS3 | KSwS1 | AStS1 | ASwS2 |
| CP8 | Diplegic | 2 | Right | 68.8 | 57.9 | 71.2 | 65.0 | KStS6 | KSwS5 | AStS4 | ASwS3 |
| CP9 | Diplegic | 1 | Left | 96.5 | 87.7 | 96.3 | 52.0 | KStS1 | KSwS0 | AStS0 | ASwS0 |
| CP10 | Diplegic | 2 | Right | 69.8 | 55.6 | 74.9 | 54.4 | KStS3 | KSwS3 | AStS3 | ASwS2 |
| CP11 | Hemiplegic left | 2 | Left | 80.3 | 69.5 | 94.3 | 62.6 | KStS6 | KSwS2 | AStS1 | ASwS0 |
| CP12 | Diplegic | 2 | Left | 74.2 | 62.0 | 85.1 | 74.0 | KStS2 | KSwS3 | AStS0 | ASwS0 |
| CP13 | Hemiplegic left | 1 | Left | 88.3 | 72.2 | 101.2 | 55.1 | KStS1 | KSwS0 | AStS0 | ASwS2 |
| CP14 | Diplegic | 2 | Right | 78.5 | 74.9 | 83.1 | 69.1 | KStS2 | KSwS0 | AStS4 | ASwS3 |
| CP15 | Hemiplegic left | 1 | Left | 79.0 | 68.4 | 91.2 | 63.3 | KStS0 | KSwS0 | AStS0 | ASwS1 |
| CP16 | Diplegic | 1 | Right | 50.4 | 34.0 | 65.0 | 63.8 | KStS6 | KSwS2 | AStS3 | ASwS2 |
| CP17 | Diplegic | 2 | Left | 68.6 | 54.3 | 84.5 | 78.9 | KStS6 | KSwS0 | AStS1 | ASwS2 |
| CP18 | Diplegic | 2 | Left | 59.8 | 41.3 | 73.6 | 66.6 | KStS6 | KSwS2 | AStS4 | ASwS3 |
| CP19 | Diplegic | 2 | Left | 73.8 | 65.8 | 75.5 | 65.4 | KStS5 | KSwS2 | AStS0 | ASwS0 |
| CP20 | Hemiplegic left | 2 | Left | 72.4 | 63.8 | 76.7 | 61.9 | KStS5 | KSwS2 | AStS0 | ASwS3 |
| CP21 | Hemiplegic right | 1 | Right | 84.7 | 76.9 | 91.8 | 60.2 | KStS6 | KSwS2 | AStS1 | ASwS0 |
| CP22 | Hemiplegic right | 2 | Right | 76.5 | 67.4 | 93.1 | 81.9 | KStS5 | KSwS2 | AStS0 | ASwS2 |
| CP23 | Hemiplegic right | 1 | Right | 74.1 | 68.0 | 87.8 | 52.8 | KStS6 | KSwS2 | AStS2 | ASwS2 |
