# Supplementary material for: A new strength assessment to evaluate the association between muscle weakness and gait pathology in children with cerebral palsy
Source: PLoS One. 2018 Jan 11;13(1):e0191097. doi: 10.1371/journal.pone.0191097 (PMC5764363; doi:10.1371/journal.pone.0191097)
Supplement: S5 Table — Passive range of motion for dorsiflexion was performed with extended knee. Spasticity is graded with the Modified Ashworth scale. For the plantar flexors, this was done with flexed- (900) and extended knee (00). Abbreviations in alphabetic order: DF = dorsiflexion; HE = hip extension; HF = hip flexion; KE = knee extension; KF = knee flexion; PF = plantar flexion; PROM = passive range of motion; SPAS = spasticity. (DOCX) [file pone.0191097.s008.docx]

| CP | Gender | Age  *years* | Weight  *kilograms* | Height  *meters* | PROM HE degrees | PROM HF  degrees | PROM KE degrees | PROM KF  degrees | PROM DF degrees | PROM PF  degrees | SPAS HF  Ashworth | SPAS KE  Ashworth | SPAS KF  Ashworth | SPAS PF 90^0^  Ashworth | SPAS PF 0^0^ Ashworth |
| --- | --- | --- | --- | --- | --- | --- | --- | --- | --- | --- | --- | --- | --- | --- | --- |
| CP1 | Girl | 12.12 | 24.2 | 1.36 | 0 | nl | 5 | nl | 5 | nl | 1 | 1+ | 1 | 1 | 1+ |
| CP2 | Boy | 12.16 | 35.7 | 1.47 | 0 | nl | 5 | nl | 5 | nl | 1 | 1 | 1 | 0 | 1+ |
| CP3 | Boy | 12.62 | 46.2 | 1.58 | 0 | nl | -5 | nl | -15 | nl | 0 | 2 | 0 | 1 | 3 |
| CP4 | Girl | 10.80 | 19.9 | 1.19 | 0 | nl | 0 | nl | 0 | nl | 1 | 1+ | 0 | 0 | 2 |
| CP5 | Boy | 11.43 | 33.7 | 1.46 | 0 | nl | 5 | nl | 10 | nl | 0 | 1 | 0 | 1 | 1+ |
| CP6 | Girl | 9.66 | 37.6 | 1.34 | 0 | nl | 5 | nl | 5 | nl | 0 | 0 | 1 | 1+ | 1+ |
| CP7 | Girl | 10.18 | 29.6 | 1.39 | -10 | nl | 0 | nl | 5 | nl | 1+ | 1+ | 1+ | 0 | 2 |
| CP8 | Girl | 9.13 | 57.5 | 1.49 | 0 | nl | 0 | nl | 15 | nl | 1 | 1+ | 2 | 0 | 2 |
| CP9 | Girl | 7.56 | 20.0 | 1.20 | 0 | nl | 0 | nl | 0 | nl | 1 | 1 | 1 | 1+ | 1+ |
| CP10 | Girl | 7.26 | 21.0 | 1.19 | 0 | nl | 5 | nl | 10 | nl | 1+ | 1 | 1+ | 1+ | 2 |
| CP11 | Boy | 8.94 | 29.0 | 1.31 | 0 | nl | 0 | nl | 20 | nl | 1 | 1+ | 1+ | 0 | 1+ |
| CP12 | Girl | 8.98 | 24.4 | 1.31 | 0 | nl | 5 | nl | 15 | nl | 1 | 1+ | 1+ | 0 | 1+ |
| CP13 | Girl | 6.96 | 23.0 | 1.22 | 0 | nl | 0 | nl | 10 | nl | 0 | 1 | 0 | 1 | 2 |
| CP14 | Girl | 14.74 | 51.7 | 1.64 | 0 | nl | 5 | nl | 5 | nl | 0 | 1 | 1+ | 0 | 1+ |
| CP15 | Boy | 12.02 | 49.8 | 1.54 | 0 | nl | 0 | nl | 0 | nl | 1 | 1+ | 0 | 1 | 2 |
| CP16 | Boy | 15.93 | 49.1 | 1.71 | -10 | nl | -25 | nl | -20 | nl | 2 | 2 | 2 | 2 | 3 |
| CP17 | Boy | 8.45 | 30.5 | 1.30 | -5 | nl | -5 | nl | -5 | nl | 1 | 0 | 2 | 0 | 1 |
| CP18 | Boy | 15.34 | 33.1 | 1.43 | -10 | nl | -20 | nl | 15 | nl | 2 | 1+ | 1+ | 0 | 1+ |
| CP19 | Girl | 9.31 | 32.6 | 1.23 | -5 | nl | 0 | nl | 5 | nl | 1+ | 1 | 3 | 1 | 3 |
| CP20 | Boy | 7.77 | 20.4 | 1.16 | 0 | nl | 10 | nl | 20 | nl | 0 | 1 | 1+ | 0 | 1 |
| CP21 | Boy | 6.51 | 22.2 | 1.20 | 0 | nl | 0 | nl | -5 | nl | 0 | N.M. | 2 | 0 | 3 |
| CP22 | Boy | 6.62 | 20.4 | 1.16 | 0 | nl | 0 | nl | 10 | nl | 0 | 0 | 1+ | 0 | 2 |
| CP23 | Boy | 11.71 | 50.6 | 1.57 | 0 | nl | 0 | nl | -5 | nl | 0 | 0 | 1+ | 0 | 2 |
