# Supplementary material for: A new strength assessment to evaluate the association between muscle weakness and gait pathology in children with cerebral palsy
Source: PLoS One. 2018 Jan 11;13(1):e0191097. doi: 10.1371/journal.pone.0191097 (PMC5764363; doi:10.1371/journal.pone.0191097)
Supplement: S6 Table — Classifications are derived from the paper of Nieuwenhuys et al: Identification of joint pattern during gait in children with cerebral palsy: a Delphi consensus study, Developmental Medicine and Child Neurology 2016, 58: 306–313 [58]. (DOCX) [file pone.0191097.s009.docx]

|  | Description of gait classification patterns |
| --- | --- |
| Knee in stance |  |
| KSTS0 | Normal knee in stance |
| KSTS1 | Increased knee flexion at initial contact |
| KSTS2 | Increased knee flexion at initial contact and earlier knee-extension movement |
| KSTS3 | Knee hyperextension |
| KSTS4 | Knee hyperextension and increased knee flexion at initial contact |
| KSTS5 | Increased knee flexion in mid-stance and internal flexion moment present |
| KSTS6 | Increased knee flexion in mid-stance and internal extension moment present |
| Knee in swing |  |
| KSWS0 | Normal |
| KSWS1 | Delayed peak knee flexion |
| KSWS2 | Increased peak knee flexion |
| KSWS3 | Increased and delayed peak knee flexion |
| KSWS4 | Decreased peak knee flexion |
| KSWS5 | Decreased and delayed peak knee flexion |
| Ankle in stance |  |
| AStS0 | Normal ankle motion during stance |
| AStS1 | Horizontal second ankle rocker |
| AStS2 | Reversed second ankle rocker |
| AStS3 | Equinus |
| AStS4 | Calcaneus |
| Ankle in swing |  |
| ASwS0 | Normal ankle in swing |
| ASwS1 | Insufficient prepositioning in terminal swing |
| ASwS2 | Continuous plantarflexion during swing (drop foot) |
| ASwS3 | Excessive dorsiflexion in swing |
